# Ethnobotanical survey of medicinal plants in the southern mountain area of Kunyu mountain, China

**DOI:** 10.3389/fphar.2025.1598940

**Published:** 2025-09-17

**Authors:** Zicheng Wang, Zhe Li, Zihan Xu, Xingjie Liu, Yinglin Wang, Ye Liu, Minghan Zhang, Huajuan Zhang, Xinyu Li, Jie Zhou, Jia Li, Qian Liu, Lingna Wang, Yongqing Zhang, Shaoping Wang, Ying Lin

**Affiliations:** ^1^ School of Pharmacy, Binzhou Medical University, Yantai, China; ^2^ School of Pharmacy, Shandong University of Traditional Chinese Medicine, Jinan, China; ^3^ Research and Development Center of Chinese Medicine Resources and Biotechnology, Shanghai University of Traditional Chinese Medicine, Shanghai, China; ^4^ School of Biological Science and Technology, University of Jinan, Jinan, China

**Keywords:** ethnobotany, southern mountain area of Kunyu mountain, medicinal plants, ethnopharmacology knowledge, field survey

## Abstract

**Introduction:**

The southern mountainous area of Kunyu Mountain is in the Jiaodong Peninsula of China, which is rich in medicinal plant resources. For a long time, the residents in the Southern Mountain area of Kunyu Mountain have used a variety of plants for pharmacy practice and have accumulated rich knowledge of medicinal plants. Although medicinal plants were widely used, there were no reports on the medicinal plants used by residents in the southern mountainous area of Kunyu Mountain. This study aimed to document the medicinal plants and evaluate the associated traditional knowledge possessed by residents of the southern mountainous area of Kunyu Mountain.

**Methods:**

Through face-to-face interviews with 256 residents, the species, preparation, and use of medicinal plants, and related traditional medicinal knowledge were quantitatively analyzed using the Informant Consensus Factor (FIC) and the Relative Frequency of Citation (RFC).

**Results:**

We identified 338 species of medicinal plants in this study, belonging to 87 families and 230 genera. Among these, Asteraceae was the dominant family and the whole grass was the most commonly used part for drug preparation, and decoction and oral administration were the most common preparation methods and routes of administration, respectively. In terms of utilization rate, *Crataegus pinnatifida* var. *major* (0.2), *Crataegus pinnatifida* (0.2), *Platycodon grandiflorus* (0.2), *Yulania denudata* (0.2), and *Zanthoxylum bungeanum* (0.2) had higher RFC values, and the five plants above were the most important medicinal plants used by the residents in this area. Besides, compared with China Pharmacopoeia, four new therapeutic uses of three known plants were found. Among the 16 disease categories in the International Classification of Primary Care (ICPC-2), Respiratory system diseases (FIC: 0.8), Digestive system diseases (FIC: 0.8), and General and unspecified system diseases (FIC: 0.8) were the most reported.

**Discussion:**

This study lists the species of medicinal plants on the southern mountainous area of Kunyu Mountain. It records their therapeutic uses, which could provide a reference for further chemical and pharmacological studies on medicinal plants.

## 1 Introduction

For centuries, humans have used medicinal plants to treat and prevent diseases and accumulated a rich knowledge of medicinal botany. According to the World Health Organization (WHO), approximately 80% of the world’s population depends on herbs to meet their health needs, especially in rural areas (WHO. Traditional Medicine, 2008). In developing and underdeveloped countries, traditional knowledge of medicinal plants plays a vital role in safeguarding the health of local people and promoting economic prosperity (Aziz et al., 2017; Li and Weng, 2017).

The Southern Mountain area of Kunyu Mountain is in the eastern part of the Jiaodong Peninsula (Shandong, China), at the junction of the Muping District of Yantai City and the Wendeng District of Weihai City. It is known as the ancestor of the sea Xianshan, it has distinct seasons and complex terrain, which can form a local climate and meet the needs of temperate plant growth, and is the Jiaodong Peninsula found the most medicinal plants mountain. After thousands of years of survival and development, residents have accumulated plentiful traditional knowledge of plant ethnomedicine, which has greatly impacted their life and health. Research on medicinal plants and their traditional uses has been increasing worldwide, and the sustainable utilization of wild medicinal plant resources is threatened (Lin et al., 2021). However, most medicinal knowledge is usually passed down from generation to generation through oral communication by residents of most rural or mountainous areas, and there is no comprehensive record of this knowledge. Additionally, with the development of society, an increasing number of young people prefer to settle in cities and are not interested in traditional medical knowledge (Hu et al., 2020). The decline in the elderly population has also aggravated the loss of ethnopharmacology knowledge from oral tradition. Consequently, it is necessary to obtain all information on the reserve and utilization status of the wild medicinal plants in the Southern Mountain area of Kunyu Mountain accurately and investigate and document information on medicinal plants and related traditional knowledge. In addition, previous studies have rarely involved an analysis of the Relative Frequency of Citation of medicinal plants. Therefore, this present study is the first to conducted a more comprehensive investigation of medicinal plants in the southern mountainous area of Kunyu Mountain, assessed the reserve and utilization status of the wild medicinal plants, recorded the medication information of interviewees, and conducted a Relative Frequency of Citation analysis to obtain a more comprehensive knowledge of local ethnopharmacology and provide ideas for related ethnopharmacology research and new drug development.

Due to the lack of a comprehensive investigation of the local ethnopharmacological system, especially the deficiency of quantitative index evaluation (Ma, 2021), we conducted an ethnopharmacology study in the Southern Mountain area of Kunyu Mountain. This study aimed to 1) investigate and classify the existing medicinal plants in the Southern Mountain area of Kunyu Mountain; 2) record the species, preparation, and use of medicinal plants and the related local knowledge; and 3) assess the consistency of the use of traditional medicines in the treatment of diseases by locals. The findings will fill the gap in the ethnobotanical knowledge of medicinal plants in the Southern Mountain area of Kunyu Mountain and provide a reference for the further study of modern medicinal plant chemistry and pharmacology.

## 2 Materials and methods

### 2.1 Description of the study area

**FIGURE 1:**
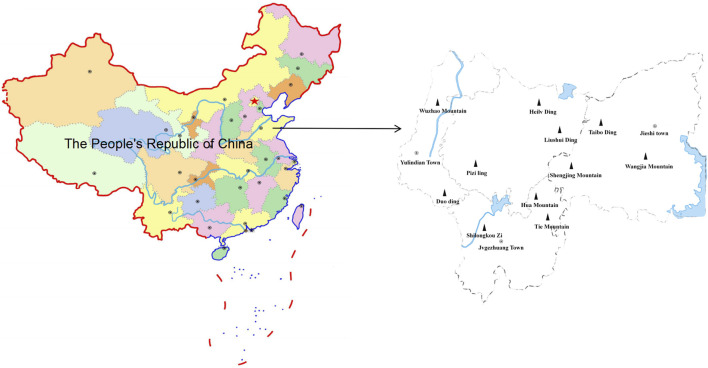
Map of the study area (Southern Mountain area of Kunyu Mountain).

The Southern Mountain area of Kunyu Mountain is situated between 121°37′0″to 121°51′0″E and 37°14′49″to 37°18′50″N (Figure 1). The strike of the mountain is Northeast-Southwest, of which the North-South length is 35 km and the East-West length is 50 km; the total area is 15,416.5 km^2^ (Fang et al., 2018). The Southern Mountain area of Kunyu Mountain is in the warm temperate monsoon climate and has four distinct seasons. The annual average temperature is 11.9 C. The average annual precipitation is 984.4 mm, mainly from June to September, accounting for 72.6% of the annual precipitation, and the frost-free period per year is approximately 200 days (Wen, 2018). The study area was the Southern mountainous area of Kunyu Mountain, extending from the Muping District of Yantai to the Wendeng District of Weihai. The research area spans three towns, including Yulindian and Jvgezhuang in the Muping District of Yantai and Jieshi town in the Wendeng District of Weihai. The terrain is mainly mountainous and hilly. There are 119 villages in the three towns surveyed in this study. By the end of 2023, the southern mountainous area of Kunyu Mountain had approximately 67,000 permanent residents. There are 37 ethnic groups, of which the Han nationality accounts for about 99.6% of the total population. The level of development of communities corresponds with the level of development of local medical facilities. Jieshi town has two local health centers, one fitness and leisure center, and 65 village health service points, with an average of one village health service point per village. Yulindain and Jvgezhuang towns have 13 and 12 health rooms, respectively, with one health center each (Muping District People’s Government website, Wendeng District People’s Government website).

**FIGURE 2:**
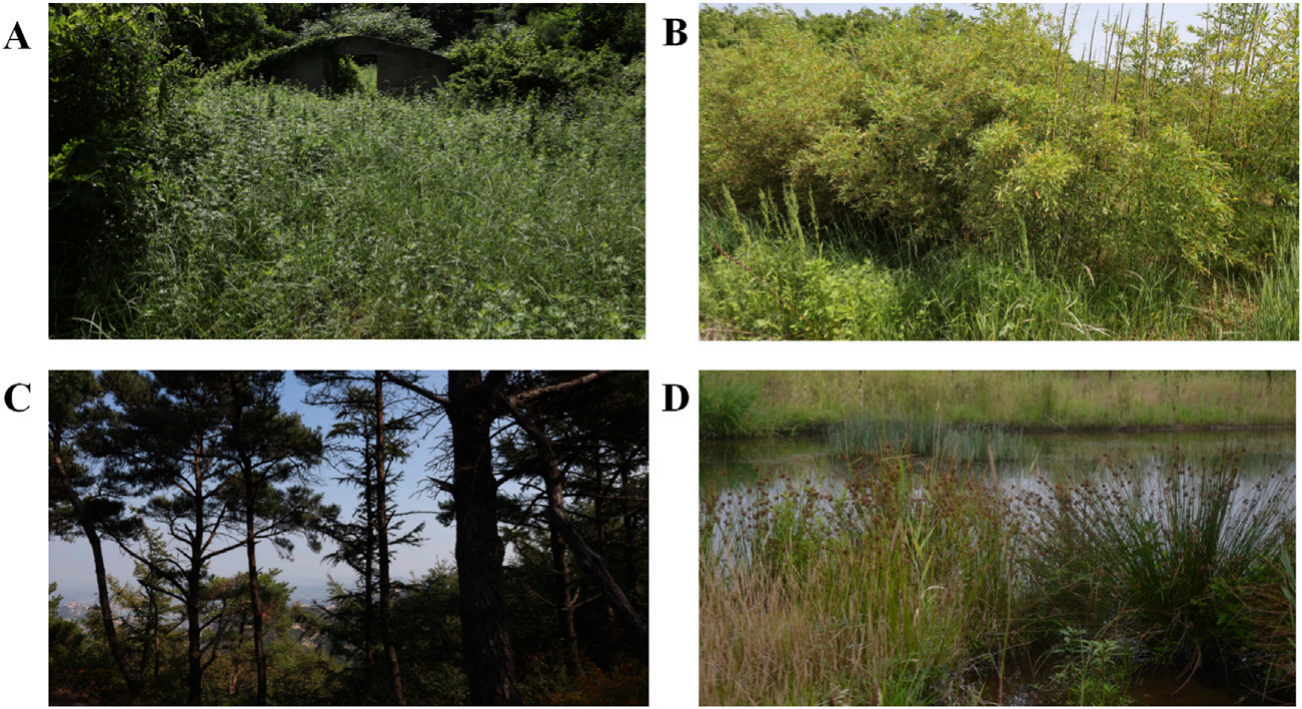
Types of vegetation in southern mountain area of Kunyu Mountain. **(A)** Grass, **(B)** Shrub, **(C)** Forest, **(D)** Wetland.

The Southern Mountain area of Kunyu Mountain has a superior geographical location and convenient transportation. Railways and expressways run through the Jiaodong Peninsula. The airport, railway station, and bus station can be reached within 1 hour. In addition, there are many open ports near the study area, and a 2-h drive can reach five national first-class open ports and three international airports. The main peaks are in the East-West area, and the highest altitude (in Jieshi Town) is 922.8 m, with a relative height difference of nearly 900 m. This area has three landforms: low mountains, hills, and plains. The main soil type is forest brown soil, followed by common brown soil. Kunyu Mountain is the source of the Hanshui, Qinshui, Muzhu, and Huanglei rivers (Wendeng District People’s Government Website). The ecosystem can be divided into forests, shrubs, arbors, and wetlands (Figure 2), and its unique ecosystem has led to different types of vegetation (Chen et al., 2022).

### 2.2 Data collection

We conducted a questionnaire survey among 256 residents (138 males and 118 females) in the Southern Mountain area of Kunyu Mountain from March 2023 to June 2024, all the respondents were selected at random. Ethnobotanical information on local plants was collected through face-to-face interviews using standardized semi-structured questionnaires (Hong et al., 2015; Teklay et al., 2013). In the survey, basic information such as sex, age, educational background, and the occupation of the respondents were collected. Concurrently, the detailed ethnobotanical information, such as the name of the plant, efficacy, and the parts of the plants used, provided by these participants, were investigated, and the detailed questions of the questionnaire are listed in Appendix A. The questionnaire was in Chinese, and all the respondents could read and complete the questionnaires. Before the interview, all respondents verbally provided informed consent, and the ethical guidelines set by the International Society for Ethnobiology (International Society of Ethnobiology, 2006) were followed. The diseases treated were classified according to the World Health Organization’s International Classification of Primary Care (ICPC-2) (International Classification Committee of WONCA, 2015).

### 2.3 Plant materials

In this study, field visits were conducted with the interviewees, and four to six medicinal plant specimens were collected based on their descriptions, with photographs of each specimen collected concurrently. Information about each plant specimen, such as the name, collection number, and collection date, was recorded on the collection label at the time of collection. Typical specimens with healthy growth were selected during collection, and intact plants with reproductive parts, vegetative parts, or other key features were preferentially selected. Additionally, bald branches, sprouting branches, and moth-eaten and physically damaged plants were avoided (Duran-Flores and Heil, 2016). Two to three pieces of blotting paper were placed above and below each specimen, and 30–40 specimens with blotting paper were placed in the specimen holder and tightly bound with a rope. The specimens in the specimen holder were completely dried in an oven at 60 C. The specimens were then frozen in a refrigerator at −80 C for 7 days to kill parasite eggs and bacteria completely. The processed dry specimens were sewn onto cardboard. Meanwhile, the key parts, such as branches, compound leaves, flowers, and fruits, were reinforced and covered with sulfate paper. Specimen identification and pressing were performed on the same day (Griffiths and Males, 2017; Sen and Samanta, 2015). The person collecting the specimen accurately recorded the collection number, the plant habitat, the plant form, and other detailed information on the collection information label. The scientific name, Chinese scientific name, family name of the plants, and identifier named the plant on the identification label (Al-Fatimi, 2019). The collection information and identification labels were pasted on the upper left and lower right corners of the cardboard, respectively (Dalar et al., 2018; Khalid et al., 2017). The identified plant specimens are preserved in the herbarium of the School of Pharmacy, Binzhou Medical University.

### 2.4 Identification of plants

The family and scientific names of medicinal plants were determined using reference books such as Flora of China (Editorial Committee of Flora of China, 1989-2013), Chinese Pharmacopoeia (Chinese Pharmacopoeia Commission, 2015), Essentials of Shandong Plants (Li, 2004), Medica Flora of Shandong (Li et al., 2013), Botany Field Practice Guide (Zhao, 2009), and Compilation of China’s Medicinal Herbs (Xie, 1996). The life forms, medicinal parts, and efficacy of medicinal plants were recorded, and the list of wild medicinal plant resources in the Southern Mountain area of Kunyu Mountain was summarized. The collected plant specimens were identificated by Ying Lin and Jia Li. The specimen number was composed of collection date and serial number + BY (Abbreviation of the name of Binzhou Medical University). Finally, the scientific names of the plant samples were checked on the world flora online website (accessed on 23 August 2024, https://www.worldfloraonline.org/).

### 2.5 Data analysis

The data collected in this study were analyzed using Microsoft Office Excel (2021) spreadsheets and SPSS version 26 software. Quantitative data analysis was conducted using the informant consensus factor (FIC) method and the number of citations.

Informant Consensus Factor: FIC = $\frac{\left(\text{Nur}-\text{Nt}\right)}{\left(\text{Nur}-1\right)}$ , where Nur is the number of individual reports of a plant for a particular disease category, and Nt is the total number of species used by all reporters for that disease category (Trotter and Logan, 1986). FIC can also reflect the importance of specific plants in the treatment of specific types of diseases in specific populations.

Relative Frequency of Citation: RFC = $\frac{\text{FC}}{N}$ s

FC refers to the number of information providers for a particular plant. N is the total number of information providers. This index indicates the local importance of a medicinal species by indicating the citation frequency. The higher the RFC, the more important a medicinal plant in a particular area. (Tardío and Pardode-Santayana, 2008).

## 3 Results

### 3.1 Demographic features of the respondents

We interviewed 256 respondents in this study (Table 1). There were 138 males (53.9%) and 118 females (46.1%). Among these, 55.5% were between 31 and 50 years old, 31.2% were older than 50, and 13.3% were between 20 and 30. The education level of the respondents was literacy (3.9%), primary school (6.6%), middle school (27.0%), high school (50.8%), and university (11.7%). The above results indicate that the residents are generally well-educated.

**TABLE 1:** Demographic features of the respondents.

Demographic features	Number	Proportion (%)
Age		
20-30	34	13.3%
31-50	142	55.5%
50 and above	80	31.2%
Sex		
Male	138	53.9%
Female	118	46.1%
Educational level		
Literate	10	3.9%
Primary school	17	6.6%
middle school	69	27.0%
High school	130	50.8%
University	30	11.7%
Occupations		
Farmer	112	43.7%
Government staff	10	3.9%
Housewife	56	21.9%
Modern medical worker	10	3.9%
Office staff	12	4.7%
Retiree	13	5.1%
Students	20	7.8%
Traditional healer	23	9.0%

Among the 256 respondents, the majority were farmers, accounting for 43.7%. The second most common occupation was housewives, accounting for 21.9%. These two occupations accounted for the largest proportion, with 168 people. Farmers and housewives knew more about medicinal plants, probably because they were involved in agricultural production and family primary medical practice, and they provided many medicinal plants. Among these, one farmer provided the most medicinal plants (56 species). Among the 256 respondents, 23 were traditional healers, five of whom obtained the traditional medical knowledge by family inheritance while the remaining 18 traditional therapists obtained the traditional medical knowledge by university studies. One traditional healer provided the most medicinal plants, with 89 species. The total number of government workers, office workers, and retirees was 25. These respondents had better traditional medical knowledge. They learned about medicinal plants from television, the media, and books. Retirees were willing to pay more attention to this kind of knowledge, one of whom provided 12 medicinal plant species. Based on the age of the respondents, young people (20–30 years old) had the least traditional medicinal plant knowledge. Twenty young people (20–30 years old) were surveyed, 12 of whom knew nothing about traditional medicine, but the remaining eight provided 10 medicinal plants. On average, each respondent in this survey provided two medicinal plants.

### 3.2 Composition of wild medicinal plant species

A total of 338 species of medicinal plants belonging to 87 families and 230 genera were recorded in this study (Supplementary Table S1). There are 328 species of angiosperms, accounting for 97.0% of all the species identified (Table 2). The total number of species that were gymnosperms, pteridophyta, and bryophyta was 10, accounting for 3.0%, Based on the plant type, 219 species, 141 genera, and 52 families were herbs, accounting for 64.8% of the total species (Table 3). Trees took the second place (58 species belonging to 43 genera and 28 families), accounting for 17.2%. Shrubs and vines were third and fourth, with proportions of 13.3% and 4.7%, respectively.

**TABLE 2:** Classification of medicinal plant in the southern mountain area of Kunyu Mountain.

Category	Habit	Number of families	Number of Genera	Number of species	Percentage of total species (%)
Angiosperms	Herb	51	138	215	63.6
	Tree	25	41	52	15.4
	Shrub	18	32	45	13.3
	Vine	10	14	16	4.7
Gymnosperm	Tree	3	2	6	1.8
Pteridophyta	Herb	2	2	3	0.9
Bryophyta	Herb	1	1	1	0.3

**TABLE 3:** Classification of large families (>10) of wild medicinal plants on the southern mountain area of Kunyu Mountain.

Family name	Species number	Total proportion (%)
Asteraceae	45	13.3
Leguminosae	22	6.5
Rosaceae	18	5.3
Labiatae	16	4.7
Polygonaceae	15	4.4

According to family classification, 45 species (13.3%) belonged to the Asteraceae family, followed by Leguminosae with 22 species (6.5%), Rosaceae (18 species, 5.3%), Labiatae (16 species, 4.7%), and Polygonaceae (15 species, 4.4%), while the 82 remaining families (65.7%) had between 1 and 10 species in each (Supplementary Table S1 and Table 3).

### 3.3 Analysis of medicinal parts of wild medicinal plants

The medicinal parts of the plants in the Southern Mountain area of Kunyu Mountain are shown in Table 4. The medicinal parts of the plants recorded in the Southern Mountain area of Kunyu Mountain included whole plant, roots and rhizomes, stems, fruits, seeds, leaves, barks, and flowers. The whole grass was the most used medicinal part (125 plants, 37.0%), followed by root and rhizome, with 96 species (28.4%), Stem (20.4%) Fruits and seeds (15.4%), Leaf (10.6%), Bark (8.6%) and Flower (8.3%). Some used parts were lower than those, such as Bulb, Thorn, Tuber, cortex, Persistent Calyx, Leaves with galls and Pollen slightly use less than 1.0%.

**TABLE 4:** The medicinal parts of wild medicinal plants on the southern mountain area of Kunyu Mountain.

Medicinal parts	Quantity	Proportion (%)
Whole plant	125	37.0
Root and rhizome	96	28.4
Stem	69	20.4
Fruits and seeds	52	15.4
Leaf	36	10.6
Bark	29	8.6
Flower	28	8.3
Other	25	7.4

### 3.4 Preparation methods and applications

A total of 12 different drug preparation methods were recorded in this study, among which decocting (301) and crushing (98) were the most commonly used preparation methods by residents, followed by juicing (14), raw (12), blending (11), alcohol maceration (10), boiling (8), vinegar (3), vinegar scalding (2), ginger (2), dipping (1), and honey burned (1) methods (Table 5). In addition, the most common route of administration was oral (322 plants), followed by topical (193 plants) (Supplementary Table S1).

**TABLE 5:** Statistics on the preparation methods of medicinal plants in Kunyu Mountain.

Methods of preparation	Number of species	Percentage of total species (%)
Decocting	301	89.1%
Crushing	98	29.0%
Juicing	14	4.1%
Raw	12	3.6%
Blend	11	3.3%
Alcohol maceration	10	3.0%
Boiling	8	2.4%
Vinegar	3	0.9%
Vinegar scald	2	0.6%
Ginger	2	0.6%
Dipping	1	0.3%
Honey burned	1	0.3%

### 3.5 Relative frequency of citation of medicinal plants

**FIGURE 3:**
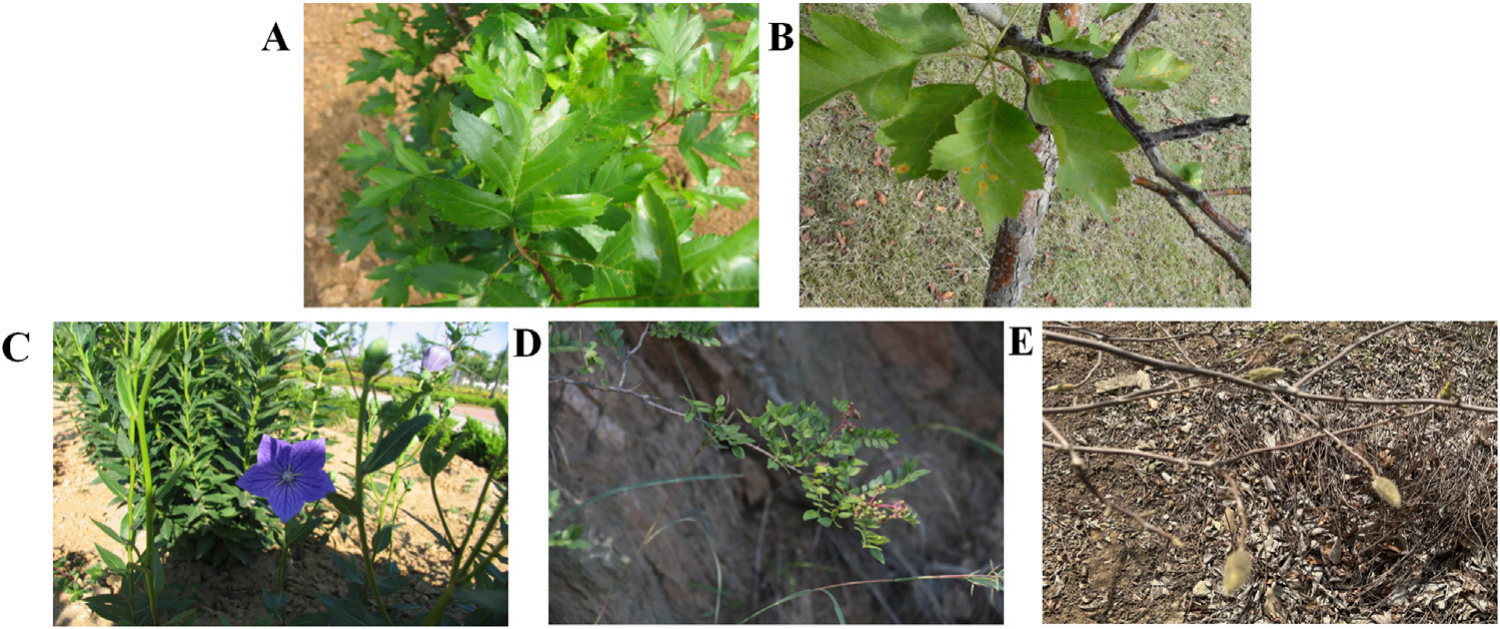
Some medicinal plants with high RFC. **(A)** The fruit trees of *Crataegus pinnatifida* var. *Major*, **(B)** The fruit trees of *Crataegus pinnatifida*, **(C)** The whole plant of *Platycodon grandiflorus*, **(D)** The fruit of *Zanthoxylum bungeanum*, **(E)** The unopened buds *Yulania denudata*.

RFC reflects the importance of medicinal plants in the study area. *C. pinnatifida* var. *major* (0.2), *Crataegus pinnatifid* (0.2), *Platycodon grandiflorus* (0.2), *Yulania denudata* (0.2), *Zanthoxylum bungeanum* (0.2) were the most useful plants on the southern mountainous area of Kunyu Mountain as indicated by their relatively high RFC (Figure 3).

*C. pinnatifid*, the plant of Rosaceae, together with its varietas, *C. pinnatifida* var. *major* both common medicinal and edible homologous plants. According to China Pharmacopoeia, the dried and mature fruits of *C. pinnatifida* var. *major* and *C. pinnatifida* can treat conditions such as Dyspepsia (D07), Loss of appetite (T03), and Hypertension (K86) (Table 2). Moreover, the two species are important income sources and are often collected and trafficked by residents from August to October. The fruits of these plants are common commodities in local markets. Regarding their medicinal applications, residents on the southern slope of Kunyu Mountain use different preparation methods to treat different diseases. For example, residents treat diarrhea using their stir-fried fruits, while the burnt fruits are used to stop bleeding.

*P. Grandiflorus* and *Z. Bungeanum* are also medicinal and edible homologous plants. According to the Chinese Pharmacopoeia, *P. grandiflorus* can be used to treat ailments such as Cough (R05) and Phlegm (R25). *Z. Bungeanum* medicinal preparations can treat Cough (R05) and Abdominal pain (D02). The survey found that 2-year-old *P. grandiflorus* was generally used for food, and more than 3-year-old *P. grandiflorus* was used as medicine by the residents of the southern mountainous area of Kunyu Mountain. Regarding food, *P. grandiflorus* can be used for cold dishes, and tender stems and leaves can also be used for cooking. The dried fruits of *Z. Bungeanum* have a wide range of uses, such as spice, seasoning, and medicinal uses.

*Y. denudata* has been cultivated in China for over 2,500 years, and its unopened buds are famous medicines for treating Sneezing or nasal congestion (R07). In addition, the flower buds of *Y. denudata* are also valuable spices and chemical raw materials that can be extracted and prepared into an essence or extract (Lu, 2017).

### 3.6 Conditions, diseases treated, and informant consensus factor (FIC)

The plants collected in this study could treat 81 diseases, divided into 16 categories based on the ICPC-2 (Supplementary Table S1 and Table 6). The FIC values for these disease categories ranged between 0.7 (circulation and skin) and 0.9 (ear), with the number of usage reports per category ranging from 7 to 876. The Ear (H) system diseases had the highest FIC value of 0.9 with two species used, On the other hand, we found that Psychological (P) system diseases, Endocrine, metabolic, and nutritional (T) system diseases and Pregnancy, child-bearing, family planning (W) system diseases were recorded to have the second, third, and fourth highest FIC values. The respiratory (R) system diseases (876 use reports, 152 plants) had the largest number of usage reports, followed by the digestive (D) system diseases (861 use reports, 211 plants), the general and unspecified (G) system diseases (790 use reports, 197 plants), and the skin (S) system diseases (410 use reports, 119 plants). These four system diseases accounted for the largest number of plant groups and usage reports, and their FIC ranged from 0.7 to 0.8.

**TABLE 6:** FIC values for the medicinal plants in the southern mountain area of Kunyu Mountain.

Diseases	Use reports (Nur)	Number of taxa (Nt)	FIC
A: General and unspecified	790	197	0.8
B: Blood, blood-forming organs, and immune mechanism	7	2	0.8
D: Digestive	861	211	0.8
F: Eye	70	19	0.7
H: Ear	10	2	0.9
K: Circulatory	89	27	0.7
L: Musculoskeletal	328	78	0.8
N: Neurological	64	18	0.7
P: Psychological	55	9	0.9
R: Respiratory	876	152	0.8
S: Skin	410	119	0.7
T: Endocrine, metabolic, and nutritional	55	9	0.9
U: Urological	184	48	0.7
W: Pregnancy, child-bearing, family planning	27	5	0.9
X: Female genital system (including breast)	236	55	0.8
Y: Male genital system	57	14	0.8

### 3.7 Comparison with regional and national medicinal plant literature

This study identified 338 medicinal plants from the Southern Mountain area of Kunyu Mountain. The results of the comparison of 14 medicinal plant works of literature are shown in Supplementary Table S1. Among them, Flora of China, Higher Plants of China, Illustration of Higher Plants of China, and Pharmacopoeia of the People’s Republic of China provide comprehensive data on the national survey or utilization of medicinal plants in China. The remaining 10 works of literature provide comprehensive data on investigating or utilizing ethnic medicinal plants in surrounding areas or the Shandong Province. Flora of China contains 328 species of medicinal plants, accounting for 97.04% of the total plants recorded (Editorial Committee of Flora of China, 1989 – 2013). Higher plants of China included 256 species of medicinal plants, accounting for 75.7% (Fu, 2012). Illustration of Higher Plants of China included 249 species of medicinal plants, accounting for 73.7% (Huang et al., 2017). There were 61 species of medicinal plants recorded in the Pharmacopoeia of the People’s Republic of China, accounting for 18.1% (Chinese Pharmacopoeia Commission, 2015). A comparison with regional and national medicinal plant literature revealed that all the medicinal plant species surveyed had already been recorded. The applications of these medicinal plants were documented in the literature, as shown in the last column of Supplementary Table S1. The result above indicates that the local medicinal plant resources were rich, and the knowledge of traditional medicinal plants was well transmitted across generations. The Southern mountainous area of Kunyu Mountain is a treasure trove for performing ethnopharmacological research.

Among the 61 species of medicinal plants recorded in the People’s Republic of China Pharmacopoeia, the following three plants have different uses: *D. indicum* for Hypertension (K86) (1 use reports), *Platycodon chinensis* for Gastroenteritis (D73) (3 use reports), and *Zanthoxylum bungeanum* for Toothache (D19) (6 use reports) and Hernia (D91) (5 use reports).

## 4 Discussion

### 4.1 Composition of wild medicinal plant species

A total of 338 species of medicinal plants belonging to 87 families and 230 genera were recorded in this study with a dominance of Asteraceae, Leguminosae, Rosaceae, Labiatae, and Polygonaceae. The results of this study show that Compositae is the dominant family, and 45 medicinal plants are Compositae plants (13.3%) in the southern mountain area of Kunyu Mountain. Asteraceae is the largest family of angiosperms, and the plants in this family have diverse reproductive modes (Lin, 1997; Hao, 2008). Furthermore, the chemical constituents of plants of the Asteraceae family are complex and diverse, including almost all types of natural compounds (Li et al., 2002), such as alkaloids with bactericidal and bacteriostatic effects; sesquiterpene lactones with antifeedant, growth-inhibiting, and toxic effects on insects; and alkyne compounds with photoactivated toxic effects on various biological organisms (Le, 1997). The above reasons contributed to the high adaptability of the plants of Asteraceae to the environment and probably account for why Asteraceae was the largest family of medicinal plants on the Southern Mountain area of Kunyu Mountain.

In terms of plant types, we found that most of the plants in the area have herbaceous habit, with trees, shrubs and Bryophyta following. Herb plants are the secondary forms with shorter life cycles and can adapt quickly to environmental changes (Wang and Jiao, 2024). Since most Pteridophyta and Bryophyta prefer humid environments and the local climate is arid with a warm temperate continental monsoon climate, Pteridophyta and Bryophyta were scarce, which also suggests that herbs are better suited to local growth than medicinal trees, shrubs and ferns.

### 4.2 Analysis of medicinal parts of wild medicinal plants

There are 14 species of plant parts that can be used as medicine in the Southern mountain area of Kunyu Mountain. We found that whole plant, roots, and rhizomes two types of medicinal parts accounted for more than 65.0% of the medicinal parts of the investigated plants, indicating that whole plant, roots, and rhizomes were the most used medicinal parts in the locality. The efficacy of different parts of the same plant might be the same or similar, but different parts of some species have different efficacy and use. Based on the efficacy and application, it is important to use different parts as drugs to treat different diseases (Huang, 2023). For example, the flowers of *L*. *japonica* can treat diseases such as sore throat (R21), cough (R05), and erysipelas (S76), while the stems and leaves can treat scabies (S72), skin diseases (S99), and musculoskeletal diseases (L99). The number of medicinal plants in the market is increasing. However, medicinal parts, such as whole grass, roots and rhizomes, and stems, require removal of the entire plant, which is more harmful for the plants than when leaves, flowers, and fruits are required. Therefore, it was more destructive to its wild resources. Especially for medicinal plants such as Orchidaceaeor and plants of other families with small population sizes, long growth cycles, and habitat fragmentation, excessive once and for all harvesting would inevitably lead to the extreme shrinkage of medicinal plant resources (Zhang et al., 2024). Therefore, it was necessary to formulate different harvesting periods for different medicinal parts to protect the long-term sustainable development of wild medicinal plant resources (Ma, 2021).

### 4.3 Preparation methods and applications

The results showed that there are 12 different methods of preparing medicines locally including Decocting, Crushing, Juicing, etc., Decocting is the preferred method by the local people which is made by steeping and decocting the herbs. Decoctions have a long history of use and the widest range of applications in China, with the advantages of easy preparation, fast absorption and the ability to quickly bring out the effects of herbs. However, different methods of preparation were used to treat different diseases. For example, the root of *P*. *multiflor*um Thunb can be used to treat malaria without preparation; however, the root above can be processed with black bean juice and used to treat anemia (Chinese Pharmacopoeia Commission, 2015).

In the local route of administration, we also found that oral ingestion was the most frequently used mode of administration, consistent with the results of previous studies (Yebouk et al., 2020; Huang et al., 2021). However, different routes of administration can treat different diseases, For example, as *Platycodon perfoliatum*, oral administration can upper respiratory infectionI acute, while external application can treat insect bites.

### 4.4 Comparison with regional and national medicinal plant literature

Interestingly, we found four new therapeutic uses for the 61 plants known by comparing them with the Chinese Pharmacopoeia. The local *D. indicum* was commonly used for hypertension, and that the *P. chinensis* was used for Gastroenteritis, whereas previous reports of *D. indicum* were mainly used to treat fever and eye pain (Wang et al., 2022). *P. chinensis* were mainly used to treat hemorrhoids and Dysentery NOS (Chinese Pharmacopoeia Commission, 2015). We also found that the local population used *Z. bungeanum* to treat toothache and hernia, but the related pharmacological research and mechanism research have not been reported, and further research is needed. which is the first reported, the plant was previously reported to be used primarily for the treatment of digestive disorders, gastrointestinal disorders, venereal diseases, respiratory disorders (Mutinda et al., 2023).

### 4.5 Conditions, diseases treated, and informant consensus factor (FIC)

The high usage rate also showed which diseases were common in the Southern Mountain area of Kunyu Mountain. For example, the top three cases in the general and unspecified (A) group were fever (A03) (53 species, 257 usage reports), bleeding (A10) (58 species, 239 usage reports), and swelling NOS (A08) (47 species, 164 usage reports), The plant species and utilization reports of these three diseases accounted for 80.2% and 83.5% of the total plant species and utilization reports of the general and unspecified (A) system diseases, respectively, also indicating that the local residents had high consistency in the treatment these three diseases. The high FIC value indicates that residents had a good acceptance and consensus on medicinal plant species and treatment methods for most diseases (Yebouk et al., 2020). However, the use of plant groups and the number of reports in some categories were small and may be misleading (Ginko et al., 2023). For example, the FIC value of the ear (H) system diseases were the highest (0.9); however, there were only 10 reports of use and only two plant species, resulting in a large FIC value. Therefore, these medicinal plants did not represent those mainly used to treat these diseases. A similar situation exists in the psychological (P) system diseases (FIC: 0.9), the endocrine, metabolic, and nutritional (T) system diseases (FIC: 0.9), and the pregnancy, childbearing, and family planning system diseases (FIC: 0.9). Based on the survey, 307 plant species can be used to treat more than one disease. For example, *L. japonicus* can treat intermenstrual bleeding, swelling NOS and urination problems diseases. 31 plants were recorded to treat only one disease. For example, *L. concolor*, *L. lancifolium*, *A. stricta*, *D. sessile*, and *L. virginicum* were recorded locally only for the treatment of coughs. Similarly, *V. collina*, *V. philippica*, and *A. shikokiana* were used to treat fever.

### 4.6 Limitations

This study inevitably has some limitations. For example, data collection on medicinal plants may not be comprehensive enough. Some steep cliffs were difficult to reach, and locals rarely utilized the medicinal plants on these cliffs; therefore, those medicinal plants could not be effectively collected. This may lead to an incomplete investigation of local medicinal plants. This is a key issue to be addressed in future research.

## 5 Conclusion

The results also showed that the reserves of traditional knowledge were related to the respondents’ residence, occupation, and age. Rural residents had more ethnopharmacological knowledge than urban residents in the southern mountainous area of Kunyu Mountain. Compared with the elderly, young people had less traditional knowledge of medicinal plants. These findings can guide the choice of respondents in future research.

This is the first study carried out in the Southern mountainous area of Kunyu Mountain to reveal the residents’ important botanical diversity and ethnobotanical knowledge. We identified 338 species of medicinal plants belonging to 87 families and 230 genera and their indigenous therapeutic uses. These plants play an important role in the local healthcare system. In addition, this study newly recorded six new uses of five known plants, but their chemical composition and pharmacological activity need to be further studied. Furthermore, *Crataegus pinnatifida*, *C. pinnatifida* var. *major*, *P.* grandiflorus, *Yulania denudata,* and *Z. bungeanum* have high RFCs and citation frequencies, and importance in the study area. Among the 16 disease categories, Respiratory, Digestive, and General and unspecified system diseases had the largest number of reports. The study also documented the preparation methods and applications of these medicinal plants, revealing the understanding and application of ethnobotanical knowledge of the residents of the Southern Mountain area of Kunyu Mountain. In conclusion, this study recorded the ethnopharmacology knowledge in the Southern Mountain area of Kunyu Mountain, providing new targets for selecting medicinal plants in pharmacology and plant chemistry research and ideas for developing new drugs.

## Data Availability

The datasets presented in this study can be found in online repositories. The names of the repository/repositories and accession number(s) can be found in the article/Supplementary Material.

## Appendix A

1. Name, gender, age and education level of the interviewee.
2. Telephone number and address of the interviewee.
3. Iterviewee occupation and number of plants provided.
4. Interview date.
5. Local name of the plant and the disease treated by the plant.
6. Medicinal parts of plants (e.g., root, stem, flower, leaf, fruit etc.).
7. How plants are prepared for use.
8. How and when plants are used.
