# Supplementary material for: Ethnobotanical survey of medicinal plants in the southern mountain area of Kunyu mountain, China
Source: Front Pharmacol. 2025 Sep 17;16:1598940. doi: 10.3389/fphar.2025.1598940 (PMC12486596; doi:10.3389/fphar.2025.1598940)
Supplement: Supplementary file 1 [file Table1.docx]

**Supplementary Table 1**

**List of wild medicinal plants on the southern mountain area of Kunyu Mountain.**

| Plant name | Scientific name | Family/voucher specimen^a^/habitatb/habit | Part used | Preparation  method | R^c^ | Diseases treated/number  of respondents (ICPC-2) | Materia medica records^d^ |
| --- | --- | --- | --- | --- | --- | --- | --- |
| Xuancao | *Hemerocallis fulva (L.)* L. | Asphodelaceae/20230601301BY/W/herb | Root | Juicing/crushing | O/T | Bleeding A10 (1)  Urination problems U05(2) | A,B,C |
| Huanghuacai | *Hemerocallis citrina* Baroni | Asphodelaceae/20230825090BY/W/herb | Root/stem | Decocting/  crushing | O/T | Jaundice D13(2) | A,B,C,L,N |
| Yulinghua | *Styrax obassis* Siebold &  Zuccarini | Styracaceae/20230825021/BY/W/shrub | Fruit/seed | Decocting | O/T | Worms/other parasites D96(1) | C |
| Qiaobingbaqia | *Smilax stans* Maxim. | Smilacaceae/20230611420/BY/W/shrub | Stem | Crushing | O/T | Rheumatism L18(5)  Bleeding A10(3) | A,B,C |
| Baqia | *Smilax china* L. | Smilacaceae/20230601003/BY/W/shrub | Stem | Vinegar /alcohol maceration | O/T | Rheumatism L18(7)  Dysentery NOS D73(2) | A,B,C,H,J,K |
| Huadongbaqia | *Smilax sieboldii* Miq. | Smilacaceae/20230825088/BY/W/shrub | Stem | Crushing | O/T | Rheumatism L18(9)  Musculoskeletal diseases L99(6) | A,B,C,N |
| Baibeiniuweicai | *Smilax nipponica* Miq. | Smilacaceae/20230825064BY/W/herb | Rhizome | Decocting | O | Rheumatism L18(5)  Menstruation absent X05(4)  Cough R05(3) | A |
| Youbanbaihe | *Lilium concolor* Salisb.  var. *pulchellum* (Fisch.) Regel | Liliaceae/20230608298/BY/W/herb | Bulb | Honey burned | O/T | Cough R05(10) | A,B,K |
| Shandan | *Lilium pumilum* DC. | Liliaceae/20240435402/BY/W/herb | Root/stem | Boiling | O/T | Cough R05(11)  Sleep disturbance P06(10)  Swelling NOS A08(1) | A,B,C,K,N |
| Juandan | *Lilium lancifolium* Thunb. | Liliaceae/20240421505/BY/W/herb | Bulb | Boiling | O/T | Cough R05(12) | A,B |
| Shuishan | *Metasequoia glyptostroboides*  Hu et W. C. Cheng | Cupressaceae/20240431403BY/W/tree | Leaf | Decocting | O | Rheumatism L18(3)  Psoriasis/Intoxication A86(2) | A,B,C |
| Xiayezhenzhucai | *Lysimachia pentapetala*  Bunge | Primulaceae/20230601252/BY/W/herb | Whole plant | Decocting | O/T | Swelling NOS A08(2)  Jaundice D13(3)  Dysentery NOS D73(2)  Rheumatism L18(3)  Bleeding A10(4) | A,B,C,N |
| Zezhenzhucai | *Lysimachia candida* Lindl. | Primulaceae/20230601341/BY/W/herb | Whole plant | Decocting | O/T | Rheumatism L18(2) | A,B,C |
| Zhenzhucai | *Pogostemon auricularius* (L.）  Hassk. | Primulaceae/20230601130/BY/W/herb | Whole plant | Decocting | O/T | Swelling NOS A08(3)  Jaundice D13(3)  Dysentery NOS D73(3)  Rheumatism L18(5)  Bleeding A10(1) | A,K |
| Aitao | *Lysimachia clethroides* Duby | Primulaceae/20230601154/BY/W/herb | Root/stem | Decocting | O | Menstruation absent X05(4)  Animal bite S13(2) | A,B,C |
| Changpu | *Acorus calamus* L. | Acoraceae Martinov/20230601324/BY/W/herb | Rhizome | Ginger | T | Dysentery NOS D73(5)  Rheumatism L18(4) | A,B,C |
| Shuimanjing | *Pseudolysimachion linariifoli-um* subsp. *dilatatum* (Nakai &  Kitagawa) D. Y. Hong | Plantaginaceae/2023061211/BY/W/herb | Whole plant | Raw | O/T | Chronic bronchitis R79(5)  Cough R05(3) | A,B,N |
| Beishuikumai | *Veronica anagallisaquatica*  Linnaeus | Plantaginaceae/2023060012/BY/W/herb | Whole plant | Raw | O/T | Dysentery NOS D73(4)  Menstruation absent X05(4)  Hypertension K86(4) | A,C |
| Dacheqian | *Plantago major* L. | Plantaginaceae/2023060310/BY/W/herb | Whole plant | Decocting | O | Cough R05(5)  Chronic bronchitis R79(5)  Diarrhea D11(6)  Hypertension K86(5) | A,B,C,G,I,K,N |
| Cheqian | *Plantago asiatica* L. | Plantaginaceae/2023060031/BY/W/herb | Seed | Decocting | O | Cough R05(6)  Chronic bronchitis R79(5)  Diarrhea D11(3)  Hypertension K86(4) | A,B,C,G,I,J,L,N |
| Bailixiang | *Thymus mongolicus* Ronn. | Labiatae/20230608011/BY/W/shrub | Whole plant | Decocting | O/T | Fever A03(5)  Cough R05(3)  Teeth ache D19(3) | A,B,C, ,K |
| Zaosu | *Phlomoides umbrosa* (Turcz.)  Kamelin & Makhm*.* | Labiatae/20230608220/BY/W/herb | Root | Decocting | O | Fever A03(3)  Cough R05(5)  Rheumatism L18(3) | A,B,C |
| Huangqin | *Scutellaria baicalensis* Georgi | Labiatae/20230526094/BY/W/herb | Root/stem | Decocting | O | Cough R05(4)  Jaundice D13(5)  Dysentery NOS D73(2) | A,B,C,J,K,L,M,N |
| Huoxuedan | *Glechoma longituba* (Nakai)  Kupr | Labiatae/20230526324/BY/W/herb | Whole plant | Dipping | O/T | Rheumatism L18(5)  Jaundice D13(4) | A,B,C |
| Neizhexiangchacai | *Isodon inflexus* (Thunberg) Kudo | Labiatae/20230532064/BY/W/herb | Whole plant | crushing | O/T | Rheumatism L18(2)  Jaundice D13(3)  Swelling NOS A08(2) | A,N |
| Yimucao | *Leonurus japonicus* Houttuyn | Labiatae/20240421/BY/W/herb | Whole plant | Ginger | O/T | Intermenstrual bleeding X08(11)  Swelling NOS A08(4)  Urination problems U05(3) | A,G,H,I,J,K,L,M,N |
| Lizhicao | *Salvia plebeia* R. Br*.* | Labiatae/20240421132/BY/W/herb | Whole plant | Decocting | O | [Fever A03(10)](https://baike.so.com/doc/3681262-3869026.html)  Teeth ache D19(4)  Animal bite S13(2) | A,B,C,G,N |
| Diguaermiao | *Lycopus lucidus* Turcz. | Labiatae/20240413102/BY/W/herb | Whole plant | Decocting | O | Intermenstrual bleeding X08(11)  Animal bite S13(1)  Swelling NOS A08(4) | A |
| Xiangchacai | *Rabdosia amethystoides* (Benth.) Hara | Labiatae/20230825253/BY/W/herb | Stem | Decocting | O/T | Fever A03(7)  Intermenstrual bleeding X08(5)  Animal bite S13(2) | A,B,C |
| Xiakucao | *Prunella vulgaris* L*.* | Labiatae/20230532132/BY/W/herb | Stem leaf | Decocting/  crushing | O/T | Blurred vision F05(8)  Jaundice D13(6)  Hepatitis NOS D97(3)  Bleeding A10(1) | A,B,C,J |
| Baitangzishu | *Callicarpa dichotoma* (Lour.) K. Koch | Labiatae/20230514053/BY/W/shrub | Rhizome/ leaf | Decocting/  crushing | O/T | Fever A03(3)  Menstrual pain X02(3) | A,B,C |
| Jingtao | *Vitex negundo* var. heterophylla (Franch.) Rehd. | Labiatae/20230511114/BY/W/herb | Root leaf | Decocting | O | Fever A03(4)  Cough R05(4)  Rheumatism L18(5) | A,B,C |
| Duohuajingu | *Ajuga multiflora* Bunge | Labiatae/20230601061/BY/W/herb | Whole plant | Decocting/  crushing | O/T | Cough R05(4)  Bleeding A10(6)  Intoxication A86(3) | A,B |
| Lanemaoyexiangchacai | *Isodon japonicus* var.  glaucocalyx (Maximowicz) H. W. Li | Labiatae/20230603221/BY/W/herb | Whole plant | Decocting/  crushing | O/T | Fever A03(3)  Intermenstrual bleeding X08(2)  Animal bite S13(3) | A,B |
| Huoxiang | *Agastache rugosa* (Fisch. et  Mey.) O. Ktze*.* | Labiatae/20230526099/BY/W/herb | Whole plant | Decocting | O/T | Heat exhaustion A88(9)  Diarrhea D11(4) | A,B,C |
| Zuojiangcao | *Oxalis corniculata* L | Labiatae/20230601331/BY/W/herb | Whole plant | Crushing | T | Fever A03(9)  Sore throat R21 (6) | A,B,C,M,N |
| Daji | *Euphorbia pekinensis* Rupr*.* | Euphorbiaceae/20230519044/BY/W/herb | Root/stem | Decocting | O/T | Swelling NOS A08(5)  Jaundice D13(3)  Urination problems U05(3) | A,B,C,H,J,L |
| Rujiangdaji | *Euphorbia esula* L*.* | Euphorbiaceae/20230421198/BY/W/herb | Whole plant | Decocting | O/T | Rheumatism L18(2)  Menstrual pain X02(3)  Constipation D12(2) | A,B,C,M,N |
| Lvcao | *Humulus scandens* (Lour.) Merr. | Cannabaceae/20230511151/BY/W/herb | Whole plant | Decocting/  crushing | O/T | Cough R05(3)  Swelling NOS A08(3)  Urination problems U05(5)  Dysentery NOS D73(2) | A,B,C,G,N |
| Dayepo | *Celtis koraiensis* Nakai | Cannabaceae/20230512161/BY/W/tree | Root/stem | Decocting/  crushing | O/T | Fever A03(3)  Sore throat R21(4) | A,B,C |
| Dengxincao | *Juncus effusus* L*.* | Juncaceae/20230608047/BY/W/herb | Stem/whole plant | Decocting | O | Swelling NOS A08(4)  Urination problems U05(3)  Sleep disturbance P06(2) | A,C,J |
| Yege | *Pueraria lobata* (Willd.) Ohwi | Papilionaceae/20230608285/BY/W/vines | Root/stem/flower | Decocting/raw | O | Fever A03(14)  Headache N01(2)  The Back symptom L02 (4)  Diarrhea D11(3) | A,L,N |
| Cihuai | *Robinia pseudoacacia* L*.* | Leguminosae/20230511037/BY/W/tree | Flower/bark | Decocting | O | Bleeding A10(8) | A,B,C,M,N |
| Huzhizi | *Lespedeza bicolor* Turcz. | Leguminosae/20230511084/BY/W/shrub | Root/flower | Decocting | O | Fever A03(3)  Cough R05(5) | A,B,C,M,N |
| Huamulan | *Indigofera kirilowii* Maxim. ex Palibin | Leguminosae/20230526086/BY/W/shrub | Flower | Decocting /crushing | O/T | Constipation D12(6)  Sore throat R21(4)  Cough R05(6)  Haemorrhoids K96(5)  Jaundice D13(1) | A,B,C,F,H |
| Kushen | *Sophora flavescens* Alt*.* | Leguminosae/20230511121/BY/W/shrub | Root/stem | Decocting | O/T | Dysentery NOS D73(2)  Pruritus S02(3)  Jaundice D13(4)  Urination problems U05(2) | A,B,C,L |
| Xigenghuzhizi | *Lespedeza virgata* (Thunb.) DC. | Leguminosae/20230512131/BY/W/shrub | Whole plant | Decocting | O | Heat exhaustion A88(2)  Urination problems U05(2)  Malaria A73(1)  Hypertension K86(1) | A,B,C,G,K |
| Yedadou | *Glycine soja* Sieb. et Zucc. | Leguminosae/20230608284/BY/W/herb | Fruit/seed | Decocting | O | Jaundice D13(4) | A,B,C,G,K,N |
| Zisuihuai | *Amorpha fruticosa* L. | Leguminosae/20230526328/BY/W/shrub | Leaf | Decocting | O | Eczema S87 (5)  Burn/scald S14 (4) | A,B,C,F,H,N |
| Hehuan | *Albizia julibrissin* Durazz. | Leguminosae/20230825079/BY/W/tree | Flower/bark | Decocting | O | Sleep disturbance P06(6)  Blurred vision F05 (5)  Loss of appetite T03(3) | C,G,L,N |
| Xinganhuzhizi | *Lespedeza davurica* (Laxmann) Schindler | Leguminosae/20230608271/BY/W/shrub | Whole plant | Decocting | O | Fever A03(4)  Cough R05(3)  Sneezing/nasal congestion R07 (9)  Gonorrhea female X71(3) Gonorrhea male Y71(5) | A,B,C,H |
| Zaojia | *Gleditsia sinensis* Lam*.* | Leguminosae/20230601310/BY/W/tree | Thorn | Decocting/  crushing | O/T | Headache N01(4)  Cough R05(3)  Swelling NOS A08(3)  Psoriasis S91(4) | A,B,C,N |
| Zhaoxianhuai | *Maackia amurensis* Rupr. et  Maxim. | Leguminosae/20230601421/BY/W/tree | Leaf/flower | Decocting | O | Rheumatism L18(2)  Dyspepsia D07(2)  Bleeding A10(5) | A,B |
| Jianyetiesaozhou | *Lespedeza juncea* (Linn. f.)  Pers. | Leguminosae/20230601105/BY/W/herb | Whole plant | Decocting/  crushing | O/T | Diarrhea D11(2)  Bleeding A10(4) | A,B,C,N |
| Zijing | *Cercis chinensis* Bunge | Leguminosae/20230421350/BY/W/shrub | Bark/fruit/flower | Decocting/  crushing | O/T | Bleeding A10(4)  Menstrual pain X02(1) | A,B,C,N |
| Rongmaohuzhizi | *Lespedeza tomentosa* (Thunb.)  Sieb. | Leguminosae/20230519197/BY/W/shrub | Root | Decocting | O | Loss of appetite T03(1) | A,B,C,G,N |
| Changejiyancao | *Kummerowia stipulacea*  (Maxim.) Makino | Leguminosae/20230518231/BY/W/herb | Whole plant | Decocting/  crushing | O/T | Rubella A74(3)  Heat exhaustion A88(2)  Dysentery NOS D73(3)  Loss of appetite T03(1) | A,B,C,N |
| Jietetiesaozhou | *Lespedeza cuneata*  *(*Dum. -Cours.) G. Don | Leguminosae/20230601110/BY/W/shrub | Whole plant | Decocting/  crushing | O/T | Blurred vision F05 (5) | A,B,C,M |
| Dahuayewandou | *Vicia bungei* Ohwi | Leguminosae/20230601231/BY/W/herb | Whole plant | Decocting | O | Cough R05(2)  Swelling NOS A08(1)  Rheumatism L18(2) | A,B,C,N |
| Yewandou | *Vicia sepium* L*.* | Leguminosae/20230601288/BY/W/herb | Whole plant | Decocting | O | Menstruation absent X05(2)  Cough R05(1) | A,B,C |
| Lvyehuzhizi | *Lespedeza buergeri* Miq. | Leguminosae/20230601214/BY/W/shrub | Root | Decocting | O | Cough R05(2)  Menstrual pain X02(2) | A,B,C,H |
| Huai | *Styphnolobium japonicum* (L.)  Schott | Leguminosae/20230526089/BY/W/tree | Leaf/flower/cortex | Decocting | O | Hepatitis NOS D97(3)  Bleeding A10(1)  Headache N01(3)  Eye pain F01(2) | A,B,C,K,N |
| Shanhuai | *Albizia kalkora* (Roxb.) Prain | Leguminosae/20230601203/BY/W/shrub | Stem | Decocting | O | Menstrual pain X02(5)  Lymph gland(s) enlarged B02(2)  Hemorrhoids K96 (2)  Sore throat R21(9)  Worms/other parasites D96(5) | A,B,C,N |
| Waitoucai | *Vicia unijuga* A. Br. | Leguminosae/20230519242/BY/W/herb | Whole plant | Decocting/  crushing | O/T | Headache N01(2)  Abdominal pain D02(2) | A,B,C |
| Yinghongdujuan | *Rhododendron mucronulatum*  Turcz. | Ericaceae/20230519297/BY/W/shrub | Leaf | Decocting | O | Headache N01(4)  Asthma R96(4)  Cough R05(5)  Chronic bronchitis R79(6) | A,B,C |
| Dujuang | *Rhododendron simsii* Planch*.* | Ericaceae/20230513419/BY/W/herb | Flower/root/stem/leaf | Decocting/  crushing | O/T | Bleeding A10(2)  Menstruation absent X05(2)  Cough R05(1) | A,B,C,H |
| Duzhong | *Eucommia ulmoides* Oliv. | Eucommiaceae/20230512349/BY/W/tree | Bark | Decocting | O | Bruise/contusion S16 (6)  Headache N01(3)  Antepartum bleeding W03(3)  Hypertension K86(12)  Urination problems U05(4)  Menstruation absent X05(5) | A,B,K,N |
| Mufangji | *Cocculus orbiculatus* (L.) DC. | Menispermaceae/20230601171/BY/W/vines | Root/stem | Decocting/  crushing | O/T | Rheumatism L18(2)  Hypertension K86(5)  Swelling NOS A08(1) | A,B,C |
| Bianfuge | *Menispermum dauricum* DC. | Menispermaceae/20230601412/BY/W/vines | Stem | Decocting/  crushing | O/T | Sore throat R21(3)  Rheumatism L18(2)  Jaundice D13(3) | A,B,C,L,N |
| Gusuipo | *Davallia trichomanoides* Blume | Polypodiaceae/20230603542/BY/W/herb | Rhizome | Decocting/  crushing | O/T | Rheumatism L18(13)  Toothache D19(8)  Diarrhea D11(7)  Impotence NOS Y07(9) | A,J |
| Langweicao | *Pennisetum alopecuroides* (L.)  Spreng. | Poaceae/20230606210/BY/W/herb | Whole plant | Decocting | O | Cough R05(4)  Rubella A74(2)  Blurred vision F05(4) | A,C,K |
| Baimao | *Imperata cylindrica* (L.) Beauv. | Poaceae/20230519007/BY/W/herb | Root | Decocting | O | Bleeding A10(5)  Heat exhaustion A88(4) | A,F,G,H,I,J,L,N |
| Quemai | *Bromus japonicus* Thunb. ex  Murr. | Poaceae/20230526194/BY/W/herb | Whole plant | Decocting | O | Infertility Y10(2) | A,C |
| Jincao | *Arthraxon hispidus* (Thunb.)  Makino | Poaceae/20230523451/BY/W/herb | Whole plant | Decocting/  crushing | O/T | Insect bite S12(1)  Cough R05(2) | A,C |
| Qiumicao | *Oplismenus undulatifolius*  (Arduino) Beauv | Poaceae/20230519192/BY/W/herb | Whole plant | Decocting/  raw | O/T | Cough R05(2)  Vomiting blood D14 (4) | A,C |
| Luwei | *Phragmites australis* (Cav.) Trin. ex Steud. | Poaceae/20230511146/BY/W/herb | Root/stem | Decocting/  crushing | O/T | Fever A03(12)  Nausea and vomitingD10(6) | A,C,G,H,I,K,L |
| Xaiohuameicao | *Eragrostis minor* Host | Poaceae/20230825264/BY/W/herb | Whole plant | Decocting/  crushing | O/T | Fever A03(12)  Urination problems U05(1)  Bleeding A10(2) | A,B,K |
| Zhifengcao | *Eragrostis ferruginea* (Thunb.)  Beauv. | Poaceae/20230823251/BY/W/herb | Root/stem | Decocting/  crushing | O/T | Bruise/contusion S16(3) | A,B,C,K |
| Fengyang | *Pterocarya stenoptera* C. DC. | Juglandaceae/20230526070/BY/W/tree | Leaf | Decocting/  crushing | O/T | Psoriasis/Intoxication A86(2)  Worms/other parasites D96(5) | A,B,C,N |
| Hutuizi | *Elaeagnus pungens* Thunb*.* | Elaeagnaceae Juss/20230532142/BY/W/shrub | Root/leaf, /fruit | Decocting | O/T | Dysentery NOS D73(6)  Cough R05(2)  Bleeding A10(5) | A,B,C |
| Gualou | *Trichosanthes kirilowii* Maxim. | Cucurbitaceae/20230526124/BY/W/herb | Fruit/seed/cortex/root/stem | Decocting/  crushing | O/T | Fever A03(10)  Phlegm R25(7)  Constipation D12(4)  Heart disease K84(2) | A,B,C,F,H,K,L,N |
| Nangua | *Cucurbita moschata* (Duch. ex Lam.) Duch. ex Poiret | Cucurbitaceae/20230523520/BY/W/herb | Seed | Decocting/  crushing | O/T | Insect bite S12(6) | A,B,C, |
| Tiangua | *Cucumis melo* L. | Cucurbitaceae/20230521130/BY/W/herb | Seed | Decocting/  crushing/raw | O | Nausea and vomiting D10(6)  Jaundice D13(5) | A,B,C |
| Huluxian | *Funaria hygrometrica* Hedw. | Funariaceae/20230525213/BY/W/herb | Whole plant | Decocting/  crushing | O/T | Bleeding A10(9)  Rheumatism L18(2) | C,N |
| Jianhua | *Betula chinensis* Maxim. | Betulaceae/20230526165/BY/W/tree | Bark | Decocting | O | Fever A03(4)  Swelling NOS A08(1) | A,B,C |
| Zhen | *Corylus heterophylla* Fisch. ex Trautv. | Betulaceae/20230523103/BY/W/tree | Fruit | Decocting/  crushing | O | Stomach function disorder D87(3)  Cough R05(5) | A,B,C |
| Eerli | *Carpinus turczaninowii* Hance | Betulaceae/20230522310/BY/W/tree | Bark | Decocting/  crushing | O/T | Swelling NOS A08(1)  Rheumatism L18(2) | A,B,C |
| Luomo | *Cynanchum rostellatum* (Turcz.) Liede & Khanum | Apocynaceae/20230601274/BY/W/herb | Whole plant | Decocting | O/T | Lactation symptom W19(3)  Impotence NOS Y07(2) | A,B,C,N |
| Biansebaiqian | *Vincetoxicum versicolor* (Bunge) Decne. | Apocynaceae/20230601344/BY/W/shrub | Root/stem | Decocting | O/T | Fever A03(3)  Cough R05(5) | A,B,C,K |
| Baishouwu | *Cynanchum bungei Decne* | Apocynaceae/20230601223/BY/W/vines | Tuber | Decocting/alcohol maceration | O/T | [Bleeding A10(3)](https://baike.so.com/doc/5363138-5598700.html)  [Impotence NOS Y07(4)](https://baike.so.com/doc/5363138-5598700.html)  Constipation D12(3) | A,B,C,K,N |
| Xuchangqin | *Vincetoxicum pycnostelma* Kitag. | Apocynaceae/20230601274/BY/W/herb | Root/stem | Decocting | O/T | \| Rheumatism L18(5) \| \| --- \|   Headache N01(3) | A,B,C,J,L,N |
| Manshengbaiwei | *Cynanchum* versicolor | Apocynaceae/20230601364/BY/W/herb | Tuber | Decocting | O | Fever A03(3)  Bleeding A10(4) | J,N |
| Ganshanbian | *Hypericum attenuatum* Choisy | Hypericum/20230601294/BY/W/herb | Whole plant | Decocting | O/T | Bleeding A10(5)  Lactation symptom W19(5) | A,B,C |
| Sisuijinlilan | *Chloranthus fortunei* (A. Gray) Solms-Laub. | Chloranthaceae/20230601231/BY/W/herb | Whole plant | Decocting | T | Contusion S16(6) | A,B,C |
| Jituijincai | *Viola acuminata* Ledeb. | Violaceae/20230601007/BY/W/herb | Whole plant | Decocting | T | Fever A03(5)  Swelling NOS A08(5)  Cough R05(3)  Contusion S16(4) | A,C |
| Qiuguojincai | *Viola collina* Bess*.* | Violaceae/20230608336/BY/W/herb | Whole plant | Decocting | T | Fever A03(3) | A,B,C,N |
| Lieyejincai | *Viola dissecta* Ledeb*.* | Violaceae/20230608321/BY/W/herb | Whole plant | Decocting | O/T | Fever A03(3)  Swelling NOS A08(1) | A,B,N |
| Zihuadidin | *Viola philippica* Cav*.* | Violaceae/20230608341/BY/W/herb | Whole plant | Decocting | O/T | Fever A03(9) | A,B,H,J,L,N |
| Xiaohuabiandangan | *Grewia biloba* G. Don var. parviflora (Bge.) Hand.-Mazz. | Malvaceae/20230511258/BY/W/shrub | Stem | Decocting/alcohol maceration | O/T | Flatulence D08(5)  Rheumatism L18(7) | A,N |
| Duanshu | *Tilia tuan* Szyszyl. | Malvaceae/20230824012/BY/W/tree | Root | Decocting/alcohol maceration | O/T | Rheumatism L18 (5)  Bleeding A10(3)  Cough R05(6) | A,B,C |
| Shukui | *Alcea rosea* Linnaeus | Malvaceae/20230608226/BY/W/herb | Whole plant | Decocting | O/T | Fever A03(6)  Cough R05 (5) | A |
| Langzhuwasong | *Orostachys cartilaginea*  Borissova | Crassulaceae/20230519126/BY/W/herb | Whole plant | Decocting | T | Bleeding A10(8)  Dysentery NOS D73(6)  Scabies S72(5) | K |
| Feicai | *Phedimus aizoon* (Linnaeus)'t  Hart | Crassulaceae/20230825068/BY/W/herb | Whole plant | Juicing | O/T | Bleeding A10(6)  Swelling NOS A08(8) | A,N |
| Changyaobabao | *Hylotelephium erythrostictum*  (Miq.) H. Ohba | Crassulaceae/20230824031/BY/W/herb | Whole plant | Decocting | T | Bleeding A10(4)  Rheumatism L18(4)  Bruise/contusion S16(4)  Burn/Scald S14(2)  Animal bite S13(2) | A,B |
| Jiegeng | *Platycodon grandiflorus* (Jacq.) A. DC. | Campanulaceae/20230519115/BY/W/herb | Root/stem | Decocting | O/T | Fever A03(5)  Phlegm R25(18)  Cough R05(17)  Constipation D12(3) | A,B,C,I,J,K,L,N |
| Zhanzhishashen | *Adenophora divaricata* Franch.  et Sav. | Campanulaceae/20230519213/BY/W/herb | Root/stem | Decocting | O | Fever A03(6)  Cough R05(7)  Gastroenteritis D73(3) | A,B,C,K |
| Qini | *Adenophora trachelioides*  Maxim. | Campanulaceae/20230519187/BY/W/herb | Root/stem | Decocting | O/T | Cough R05(2)  Phlegm R25(2) | A,B,C,N |
| Lunyeshashen | *Adenophora tetraphylla* (Thunb.) Fisch. | Campanulaceae/20230519168/BY/W/herb | Root/stem | Decocting | O | Fever A03(4)  Phlegm R25(7)  Cough R05(5) | A,B,C,L |
| Shishashen | *Adenophora polyantha* Nakai | Campanulaceae/20230526223/BY/W/herb | Root/stem | Decocting | O | Phlegm R25(3)  Cough R05(6) | A,B,C,N |
| Shashen | *Adenophora stricta* Miq. | Campanulaceae/20230526364/BY/W/herb | Root/stem | Decocting | O | Cough R05(4) | A,B,I,J,N |
| Xiyeshashen | *Adenophora capillaris* subsp.  paniculata (Nannfeldt) D. Y. Hong & S. Ge | Campanulaceae/20230526643/BY/W/herb | Root/stem | Decocting | O | Bronchitis R78(1)  Cough R05(3)  Hypertension K86(1)  Malaria A73(1)  Menstrual pain X02(2) | A,N |
| Yewoju | *Lactuca serriola* Linnaeus | Asteraceae/20230526310/BY/W/herb | Whole plant | Decocting | O | Fever A03(2)  Animal bite S13  Cough R05(3)  Abdominal pain D02(3) | A,B |
| Aihao | *Artemisia lancea* Van. | Asteraceae/20230526235/BY/W/herb | Root | Decocting | O/T | Bleeding A10(1)  Menstrual pain X02(1) | A,B,C |
| Ai | *Artemisia argyi* Levl. et Van. | Asteraceae/20230511002/BY/W/herb | Whole plant | Decocting | O/T | Bleeding A10(3)  Menstrual pain X02(19)  Bleeding A10(8) | A,B,C,F,G,H,I,J,N |
| Bailianhao | *Artemisia stechmanniana* Bess. | Asteraceae/20230526005/BY/W/herb | Whole plant | Juicing | T | Cough R05(1)  Jaundice D13(2)  Rheumatism L18 (5)  Dysentery NOS D73(2) | A,N |
| Dadingcao | *Leibnitzia anandria* (Linnaeus)  Turczaninow | Asteraceae/20230526031/BY/W/herb | Whole plant | Juicing | T | Rheumatism L18 (5)  Cough R05(2) | A,B,C,N |
| Jianyejinjijv | *Coreopsis lanceolata* L*.* | Asteraceae/20230519107/BY/W/herb | Whole plant | Decocting | O | Contusion S16(1) | A,B |
| Jvyu | *Helianthus tuberosus* L. | Asteraceae/20230511117/BY/W/herb | Tuber | Decocting | O/T | Rheumatism L18(4)  Heat exhaustion A88(2)  EczemaS87(6)  Stomach function disorder D87 (3) | A,B,C,G,N |
| Nanmuhao | *Artemisia eriopoda* Bge. | Asteraceae/20230526174/BY/W/herb | Whole plant | Decocting | O/T | Heat exhaustion A88(2)  Headache N01(1)  Rheumatism L18(3)  Animal bite S13(1) | A,B,C,N |
| Tuersan | *Syneilesis aconitifolia* (Bge.)  Maxim. | Asteraceae/20230526135/BY/W/herb | Root/stem | Decocting | O | Rheumatism L18(4)  Malaria A73(2)  Cough R05(4)  Animal bite S13(2) | A,B,C |
| Zhumaohao | *Artemisia scoparia* Waldst. et  Kit. | Asteraceae/20230526323/BY/W/herb | Whole plant | Decocting | O | Heat exhaustion A88(2)  Rheumatism L18 (2)  Jaundice D13(1) | A,B,C,I,N |
| Linzelan | *Eupatorium lindleyanum* DC*.* | Asteraceae/20230511140/BY/W/herb | Leaf | Decocting | O | Rheumatism L18 (2)  Menstrual pain X02(5)  Swelling NOS A08(1) | A,B,N |
| Canger | *Xanthium strumarium* L. | Asteraceae/20230601023/BY/W/herb | Fruit | Decocting | O | Rheumatism L18(4)  Headache N01 (8)  Sneezing/nasal congestion R07 (9) | A,G,H,I,J,L,N |
| Cangzhu | *Atractylodes Lancea* (Thunb.)  DC. | Asteraceae/20230601136/BY/W/herb | Root/stem | Decocting | O | Rheumatism L18 (3)  Blurred vision F05 (2) | A,B,C,J |
| Yinianpeng | *Erigeron annuus* (L.) Pers. | Asteraceae/20230526290/BY/W/herb | Whole plant | Decocting | O | Dyspepsia D07(2)  Gastroenteritis D73(1)  Malaria A73(4) | A,B,C,N |
| Ji | *Cirsium japonicum* Fisch. ex  DC. | Asteraceae/20230825045/BY/W/herb | Whole plant | Juicing | T | Bleeding A10(14)  Eczema S87(4)  Intoxication A86(3) | A,B,G,I,J,L,N |
| Xipengcao | *Conyza canadensis* (L.) Cronq. | Asteraceae/20240421266/BY/W/herb | Whole plant | Decocting | O | Rheumatism L18(2)  Bronchitis R78 (3)  Swelling NOS A08(1) | A,G |
| Fenmaojv | *Saussurea japonica* (Thunb.)  DC. | Asteraceae/20240421352/BY/W/herb | Whole plant | Decocting/alcohol maceration | O | Rheumatism L18 (2)  Menstruation absent X05 (4)  Abdominal pain D02 (4) | A,B,C,N |
| Niuxijv | *Galinsoga parviflora* Cav*.* | Asteraceae/20240411234/BY/W/herb | Whole plant | Juicing | O/T | Bleeding A10(3)  Bronchitis R78(3) | A,B,C,N |
| Linyinqianliguang | *Senecio nomorensis* L. | Asteraceae/20240421341/BY/W/herb | Whole plant | Decocting | O/T | Gastroenteritis D73 (4)  Hepatitis NOS D97 (6)  Dysentery NOS D73(4) | A,B,C |
| Huanghuapoluomenshen | *Tragopogon orientalis* L*.* | Asteraceae/20230601092/BY/W/herb | Flower | Decocting | O/T | Cough R05(3) | A,B |
| Shuqucao | *Gnaphalium affine* D. Don | Asteraceae/20230601087/BY/W/herb | Stem leaf | Decocting | O/T | Cough R05 (6)  Phlegm R25 (5) | A |
| Shanwojv | *Lagedium sibiricum* (L.) Sojak | Asteraceae/20230601067/BY/W/herb | Whole plant | Decocting | O/T | Tonsillitis acute R76 (1)  Hemorrhoids K96 (1) | A,C |
| Yejv | *Dendranthema indicum* (L.) Des Moul. | Asteraceae/20230608286/BY/W/herb | Whole plant | Decocting | O | Blurred vision F05 (2)  Headache N01(6)  Hypertension K86(1) | A,I,J,L,N |
| Dongfengcai | *Doellingeria scaber* (Thunb.)  Nees | Asteraceae/20230608267/BY/W/herb | Whole plant | Decocting | O | Headache N01(6)  Blurred vision F05 (2)  Tonsillitis acute R76 (1)  Animal bite S13 (1) | A |
| Jianliejiahuanyangshen | *Crepidiastrum sonchifolium*  (Maximowicz) Pak & Kawano | Asteraceae/20230608196/BY/W/herb | Whole plant | Decocting | O | Tonsillitis acute R76 (1)  Bleeding A10 (8)  Headache N01 (1)  Toothache D19 (2) | A |
| Loulu | *Stemmacantha uniflora* (L.)  Dittrich | Asteraceae/20230511145/BY/W/herb | Root/stem | Decocting | O | Fever A03(11)  Blurred vision F05 (4)  Lactation symptom W19(3) | A,C,F,J,K,N |
| Lanhuashichejv | *Cyanus segetum* Hill | Asteraceae/20230511243/BY/W/herb | Whole plant | Decocting | O/T | Jaundice D13(1)  Rheumatism L18(2) | A |
| Junchenhao | *Artemisiacapillaris* Thunb. | Asteraceae/20230526294/BY/W/herb | Stem leaf | Decocting | O/T | Heartburn D03(4)  Jaundice D13(1)  Urination problems U05(6) | A,B,C,G,H,I,L |
| Feipeng | *Erigeron acer* Linn. | Asteraceae/20230526067/BY/W/herb | Whole plant | Decocting/  Crushing/juicing | O/T | Fever A03(2)  Tonsillitis acute R76 (1)  Palpitations K04 (1)  Hypertension K86 (6) | C |
| Chiguojv | *Pterocypsela indica* (L.) Shih | Asteraceae/20230526031/BY/W/herb | Whole plant | Decocting/raw | O | Menstruation absent X05 (1)  Rheumatism L18 (2)  Bleeding A10(3) | A,C,N |
| Mengguhao | *Artemisia mongolica*  (Fisch. ex Bess.) Nakai | Asteraceae/20230526168/BY/W/herb | Whole plant | Decocting | O | Fever A03(5)  Menstrual pain X02 (2) | A,B,C |
| Jinzhanyinpan | *Bidens biternata (Lour.)* Merr.  et Sherff | Asteraceae/20230526113/BY/W/herb | Whole plant | Decocting/alcohol maceration | O | Tonsillitis acute R76 (1)  Dysentery NOS D73(6)  Jaundice D13 (5)  Gastroenteritis D73 (1) | A,B,C,N |
| Dalihao | *Artemisia sieversiana* Ehrhart ex Willd. | Asteraceae/20230526142/BY/W/herb | Whole plant | Decocting | O | Fever A03(1)  Tonsillitis acute R76 (1) | A,B,C |
| Huayediankucai | *Sonchus asper* (Linn.) Hill*.* | Asteraceae/20230519087/BY/W/herb | Whole plant | Raw | O | Bleeding A10(6)  Eczema S87(3) | A,B,C,N |
| Lichang | *Eclipta prostrata* (L.) L. | Asteraceae/20230519063/BY/W/herb | Whole plant | Decocting/  crushing | O/T | Bleeding A10(3)  Vertiginous syndrome H82(6)  Sleep disturbance P06(3) | A,B,C,G,H,N |
| Ciercai | *Cirsium setosum* (Willd.) MB. | Asteraceae/20230511036/BY/W/herb | Whole plant | Decocting | O | Hematuria U06 (1)  Vomiting blood D14(5)  Swelling localized S04(5) | A,G,H,I,K,L,M,N |
| Kuihao | *Artemisia princeps* Pamp. | Asteraceae/20230511043/BY/W/herb | Whole plant | Decocting | O/T | Menstruation excessive X06 (5)  Rheumatism L18(3) | A,B,C |
| Huorongcao | *Leontopodium leontopodioides* (Willd.) Beauv. | Asteraceae/20230608353/BY/W/herb | Whole plant | Decocting | O | Fever A03(2)  Urination problems U05(4)  Bleeding A10(4) | A,B,C,N |
| Kumaicai | *Ixeris polycephala* Cass. | Asteraceae/20230601123/BY/W/herb | Whole plant | Decocting | O | Skin diseases S99(4)  Skin injury S19(5) | A,B,C,N |
| Nihucai | *Hemisteptia lyrata* (Bunge)  Fischer & C. A. Meyer | Asteraceae/20230601177/BY/W/herb | Whole plant | Decocting/  crushing | O/T | Skin diseases S99(5)  Rubella A74(10）  Pruritus S02 (1) | C,G,N |
| Pugongying | *Taraxacum mongolicum*  Hand.-Mazz. | Asteraceae/20240421185/BY/W/herb | Whole plant | Decocting | O | Skin diseases S99(8)  Jaundice D13(4)  Tonsillitis acute R76 (1) | A,B,C,G,H,I,J,K,L,N |
| Sanmaiziwan | *Aster trinervius* subsp. ageratoides (Turczaninow) Grierson | Asteraceae/20240421176/BY/W/herb | Whole plant | Decocting | O | Bronchitis R78(12)  Fever A03(6)  Cough R05(16) | A,K,N |
| Shanhao | *Artemisia brachyloba* Franch. | Asteraceae/20240421160/BY/W/herb | Whole plant | Decocting | O | Headache N01(4)  Sore throat R21 (6)  Rheumatism L18 (2) | A,B,C |
| Yacong | *Scorzonera austriaca* Willd. | Asteraceae/20240421278/BY/W/herb | Root/stem | Decocting | O | Skin injurys19(4)  Skin diseases S99(7) | A,B,M,N |
| Zhonghuakumaicai | *Ixeris chinensis* (Thunb.) Nakai | Asteraceae/20240421269/BY/W/herb | Whole plant | Decocting | O | Skin diseases S99(5)  Jaundice D13(5)  Urination problems U05(5) | A,B,C,N |
| Ruichihuli | *Quercus aliena* Bl. var.  acuteserrata Maxim. ex Wenz. | Fagaceae/20230843340/BY/W/tree | Bark | Decocting/  charring | O/T | Cough R05(5)  Diarrhea D11(7) | A,C |
| Shuanpili | *Quercus variabilis* Bl. | Fagaceae/20230843421/BY/W/tree | Fruit/seed | Decocting | O/T | Cough R05(6)  Diarrhea D11(3) | A,B,C |
| Hushu | *Quercus dentata* Thunb. | Fagaceae/20230842132/BY/W/tree | Bark/shell | Decocting/  charring | O/T | Hemorrhoids K96(6) Gonorrhea female X71(3) Gonorrhea male Y71(5) | A,C,K |
| Mali | *Quercus acutissima* | Fagaceae/20230601154/BY/W/tree | Bark/leaf/shell/fruit | Decocting | O | Diarrhea D11(5)  Swelling NOS A08(4) | A,B,C,N |
| Duanbingpaoli | *Quercus glandulifera* Bl. | Fagaceae/20230519059/BY/W/tree | Leaves with galls | Decocting | O | Abdominal pain epigastric D02(2)  Urination problems U05(5) | A,B |
| Huli | *Quercus aliena* Bl. | Fagaceae/20230519063/BY/W/tree | Bark/shell | Decocting/  charring | O | Bleeding A10(3)  Diarrhea D11(6) | A,B,C |
| Li | *Castanea mollissima* Bl. | Fagaceae/20230519134/BY/W/tree | Fruit/seed/flower/bark/shell | Boiling | O | Diarrhea D11(6)  Impotence Y07(4)  Skin diseases S99(5)  Skin injury s19(3)  Whooping cough R71(3)  Hernia D91(2) | A,B,C,M,N |
| Chouchun | *Ailanthus altissima* (Mill.)  Swingle | Simaroubaceae/20230421032/BY/W/herb | Bark/fruit | Decocting | O | Diarrhea D11(7)  Bleeding A10(8) | A,B,C,J,L,N |
| Yangersuan | *Liparis japonica* (Miq.) Maxim. | Orchidaceae/20230608214/BY/W/herb | Whole plant | Decocting | O/T | Bleeding A10(4)  Skin injury s19(6) | A,B |
| Shoucao | *Spiranthes sinensis* (Pers.) Ames | Orchidaceae/20230608362/BY/W/herb | Whole plant | Decocting | O | Bleeding A10(5)  Cough R05(2)  Diabetes T90(6) | A,B |
| Chiguosuanmo | *Rumex dentatus* L. | Polygonaceae/20230608333/BY/W/herb | Root leaf | Decocting/  crushing | O/T | Worms/other parasites D96(6)  Pruritus S02(6)  Skin diseases S99(7) | A,B,C,G,M,N |
| Gangbangui | *Polygonum perfoliatum* L. | Polygonaceae/20230601073/BY/W/herb | Whole plant | Decocting/  crushing | O/T | Cough R05(8)  Bleeding A10(4)  Animal bite S13(9)  Urination problems U05(6)  Swelling NOS A08(4) | A,B,C,G,H,I,J,L,N |
| Huzhang | *Reynoutria japonica* Houtt. | Polygonaceae/20230601501/BY/W/herb | Root/stem | Decocting | O/T | Skin injury S19(3)  Menstruation absent X05(5)  Cough R05(3)  Jaundice D13(8)  Skin diseases S99(1) | A,J,K,L,N |
| Jinqiaomai | *Fagopyrum dibotrys* (D. Don)  Hara | Polygonaceae/20230601304/BY/W/herb | Tuber | Decocting | O | Cough R05(5)  Skin diseases S99(1)  Rheumatism L18(5) | A,B,J,N |
| Suanmoyeliao | *Polygonum lapathifolium* L. var. lapathifolium | Polygonaceae/20230608233/BY/W/herb | Leaf | Decocting | O | Rheumatism L18(6)  Eczema S87(1)  Swelling NOS A08(6) | A,B,C,G,H,M,N |
| Jiyeliao | *Polygonum thunbergii* Sieb. et  Zucc. | Polygonaceae/20230601103/BY/W/herb | Whole plant | Decocting/  crushing | O/T | Diarrhea D11(5)  Animal bite S13(4) | A,B,C |
| Chafenliao | *Polygonum divaricatum* L. | Polygonaceae/20230601332/BY/W/herb | Root/stem | Decocting/  crushing | O | Diarrhea D11(5)  Abdominal pain D02(5) | A,C,M |
| Yangti | *Rumex japonicus* Houtt. | Polygonaceae/20230526282/BY/W/herb | Root | Decocting | O/T | Bleeding A10(6) | A,B,C,M |
| Quanshen | *Polygonum bistorta* L. | Polygonaceae/20230526238/BY/W/herb | Root/stem | Decocting | O/T | Bleeding A10(4)  Cough R05 (6)  Diarrhea D11(9) | A,B,J,L |
| Ciliao | *Polygonum senticosum* (Meisn.) Franch. et Sav. | Polygonaceae/20230526293/BY/W/herb | Whole plant | Decocting/  crushing | T | Eczema S87(5)  Pruritus S02(7) | A,B,C |
| Shuiliao | *Polygonum hydropiper* L. | Polygonaceae/20230526301/BY/W/herb | Whole plant | Decocting | O/T | Abdominal pain epigastric D02(3)  Rheumatism L18(6)  Diarrhea D11(7) | A,B,C,G,M |
| Heshouwu | *Fallopia multiflora* (Thunb.)  Harald. | Polygonaceae/20240421080/BY/W/vines | Tuber | Decocting | O/T | Impotence NOS Y07(9)  Anaemia B81(5)  Skin diseases S99(3)  Malaria A73(5)  Constipation D12(7) | A,H,I,J,N |
| Zhouyesuanmo | *Rumex crispus* L. | Polygonaceae/20230526321/BY/W/herb | Root | Decocting/  crushing | O/T | [Bleeding A10(6)](https://baike.so.com/doc/4183497-4383993.html)  [Constipation D12(4)](https://baike.so.com/doc/4183497-4383993.html)  [Worms/other parasites D96](https://baike.so.com/doc/4183497-4383993.html)(5) | A,B,C,M |
| Yangtisuanmo | *Rumex japonicus* | Polygonaceae/20230526130/BY/W/herb | Whole plant | Decocting | O/T | Bleeding A10 (6)  Constipation D12(3)  Swelling NOS A08(6) | N |
| Suanmo | *Rumex acetosa* L. | Polygonaceae/20230526348/BY/W/herb | Root | Decocting | O/T | Bleeding A10(3)  Constipation D12(7) Urination problems U05(6)  Worms/other parasites D96(5) | A,B,C,N |
| Yuejiancao | *Oenothera erythrosepala* Borb. | Onagraceae/20230601308/BY/W/herb | Flower/seed/Root | Decocting/  crushing | O | Rheumatism L18(5) | A,B,K |
| Machixian | *Portulaca oleracea* L. | Portulacaceae/20230825155/BY/W/herb | Whole plant | Crushing | O/T | Urination problems U05(5)  Acne S96(6)  Dysentery NOS D73(6)  Erysipelas S76(7) | A,B,C,G,H,I,J,K,L,M,N |
| Madouling | *Aristolochia debilis* Sieb. et  Zucc | Aristolochiaceae/20230825163/BY/W/herb | Root/Stem/fruit | Decocting | O | Cough R05(4)  Hemorrhoids K96(5)  Bleeding A10(4) | A,B,C,H,I,K,N |
| Laohecao | *Geranium wilfordii* Maxim. | Geraniaceae/20230511128/BY/W/herb | Whole plant | Decocting/alcohol maceration | O/T | Rheumatism L18(6)  Diarrhea D11(2)  Teeth ache D19(1)  Acne S96(4) | A,B,C,K,L,N |
| Baitouweng | *Pulsatilla chinensis* (Bunge)  Regel | Ranunculaceae/20230511009/BY/W/herb | Root | Decocting/  curshing | O/T | Hemorrhoids K96 (1)  Dysentery NOS D73(6) | A,B,C,H,I,J,K,L,N |
| Huihuisuan | *Ranunculus chinensis* Bunge | Ranunculaceae/20230601096/BY/W/herb | Whole plant | Decocting/  curshing | O/T | Malaria A73(2)  Jaundice D13(5)  Asthma R96(6) | A,B,C,G,N |
| Changdongcao | *Clematis hexapetala* var.  tchefouensis | Ranunculaceae/20240421313/BY/W/herb | Root/stem | Decocting/  curshing | O/T | Rheumatism L18(6)  Swelling NOS A08(3)  Hemorrhoids K96 (1) | A,B,N |
| Dongyatangsongcai | *Thalictrum minus* var.  hypoleucum | Ranunculaceae/20230519054/BY/W/herb | Root | Decocting | O/T | Heartburn D03(3)  Cough R05(5)  Eye pain F01(2)  Dysentery NOS D73(4) | A,K |
| Dayetiexianlian | *Clematis heracleifolia* DC. | Ranunculaceae/20230519061/BY/W/vines | Root | Decocting | O/T | Gastroenteritis D73 (4)  Dysentery NOS D73(4)  Rheumatism L18(6) | A,B,C,K,N |
| Maogen | *Ranunculus japonicus* Thunb. | Ranunculaceae/20230519063/BY/W/herb | Root/stem | Decocting/  curshing | O/T | Jaundice D13(6)  Malaria A73(4)  Rheumatism L18(6)  Asthma R96(2)  Acne S96(4) | A,B,C,N |
| Shandongyinlianhua | *Anemone shikokiana* (Makino) Makino | Ranunculaceae/20230519053/BY/W/herb | Root/stem | Decocting | O | Fever A03(3) | A |
| Baiyulan | *Yulania denudata* (Desr.) D. L. Fu | Magnoliaceae/20230519861/BY/W/tree | Flower leaf | Decocting/  curshing | O/T | Fever A03(5)  Urination problems U05(6)  Bronchitis R78(6)  Flatulence D08(7)  Cough R05(4)  Sneezing/nasal congestion R07 (6)  Headache N01(7) | A |
| Yulan | *Magnolia denudata* Desr. | Magnoliaceae/20230519634/BY/W/tree | Flower | Decocting/  curshing | O/T | Breathing problem R04(5)  Menstrual pain X02(5)  Sneezing/nasal congestion R07(6) | A,B,C |
| Ezhangqiao | *Liriodendron chinense* (Hemsl.) Sarg. | Magnoliaceae/20230519394/BY/W/tree | Root/stem/cortex | Decocting | O | Rheumatism L18(5)  Cough R05(4) | A,B,C |
| Mutong | *Akebia quinata* (Houttuyn)  Decaisne | Lardizabalaceae/20230519363/BY/W/vines | Fruit/seed/stem | Decocting | O | Urination problems U05(2)  Menstruation absent X05(4)  Heartburn D03(1) | A,B,J |
| Bailashu | *Fraxinus chinensis* Roxb. | Oleaceae/20230825169/BY/W/tree | Bark | Curshing | O | Menstruation absent X05(4)  Dysentery NOS D73(4)  Eye pain F01(3)  Blurred vision F05 (5) | A,B,C,H |
| Xueliu | *Fontanesia fortunei* Carrière | Oleaceae/20230825277/BY/W/tree | Bark | Decocting | O/T | Menstruation absent X05(2)  Menstrual pain X02(3) | A,C |
| Huaquliu | *Fraxinus rhynchophylla* Hance | Oleaceae/20230825383/BY/W/tree | Bark | Decocting | O | Eye pain F01(3)  Blurred vision F05 (5) | A |
| Lianqiao | *Forsythia suspensa* (Thunb.)  Vahl | Oleaceae/20230601135/BY/W/shrub | Fruit/seed | Boiling | O | Fever A03(3)  Erysipelas S76 (3)  Acne S96(4)  Upper respiratory infection acute R74 (7) | A,J |
| Jiejiecao | *Equisetum ramosissimum* Desf. | Oleaceae/20230526109/BY/W/herb | Whole plant | Decocting/  curshing | O | Eye pain F01(3)  Cough R05(6)  Sore throat R21(4) | A,B,C, ,G,M,N |
| Wenjing | *Equisetum arvense* L. | Oleaceae/20230421246/BY/W/herb | Whole plant | Decocting/  curshing | O/T | Fever A03(3)  Hematuria U06(5)  Cough R05(3)  Eye pain F01(6) | A,B,C, ,G,K,M,N |
| Maopaotong | *Paulownia tomentosa* (Thunb.)  Steud. | Paulowniaceae/20230526165/BY/W/tree | Root/stem/flower/leaf | Blend/Decocting | O/T | Hemorrhoids K96 (2)  Cough R05(4)  Erysipelas S76(2) | A,B,C,M,N |
| Dijin | *Parthenocissus tricuspidata*  (Siebold & Zucc.) Planch. | Vitaceae/20230526050/BY/W/vines | Whole plant | Decocting/  curshing | O/T | Bleeding A10 (2)  Jaundice D13(5)  Dysentery NOS D73(7) | A,B,C,G,J,L,N |
| Dongbeisheputao | *Ampelopsis heterophylla*  (Thunb.) Sieb. et Zucc. var.  brevipedunculata (Regel) C.L.Li | Vitaceae/20230526036/BY/W/vines | Bark | Blend/curshing | T | Vomiting D10(3)  Bleeding A10(5)  Acne S96(3) | A |
| Shanputao | *Vitis amurensis* Rupr. | Vitaceae/20230526238/BY/W/herb | Fruit/seed | Blend | O | Bleeding A10(5)  Jaundice D13 (3) | A,B,C,K |
| Sheputao | *Ampelopsis glandulosa* | Vitaceae/20230601218/BY/W/vines | Bark | Blend/Decocting | O/T | Acne S96(5)  Bleeding A10(4)  Swelling NOS A08(2) | A |
| Lvyesheputao | *Ampelopsis humulifolia* Bunge | Vitaceae/20230608152/BY/W/vines | Bark | Decocting/  curshing | O/T | Rheumatism L18 (3)  Swelling NOS A08(4) | A,B,C,N |
| Yiyedijin | *Parthenocissus dalzielii* Gagnep. | Vitaceae/20230526050/BY/W/vines | Root | Blend/Decocting | O/T | Rheumatism L18 (4)  Acne S96 (2)  Headache N01(5) | A |
| Yanfumu | *Rhus chinensis* Mill. | Anacardiaceae/20230601281/BY/W/tree | Root/stem/leaf/flower/Fruit/seed | Blend/Decocting | O/T | Asthma R96 (4)  Swelling NOS A08(3) | A,B,C |
| Huanglianmu | *Pistacia chinensis* Bunge | Anacardiaceae/20230601361/BY/W/tree | Bark/leaf | Burning/Decocting | O/T | Dysentery NOS D73(3)  Acne S96(5) | A,B,C |
| Ziwei | *Lagerstroemia indica* L. | Lythraceae/20230601391/BY/W/tree | Root/cortex/Leaf/flower | Decocting/alcohol maceration | O/T | Menstruation excessive X06 (3)  Bleeding A10(3)  Erysipelas S76(2)  Acne S96(1) | A,B,C,M |
| Zhuyangyang | *Galium spurium* L. | Rubiaceae/20240421324/BY/W/herb | Whole plant | Blend | T | Swelling NOS A08(4)  Menstrual pain X02(2)  Acne S96(4) | H,I, |
| Qiancao | *Rubia cordifolia* L. | Rubiaceae/20230511190/BY/W/herb | Root | Decocting/alcohol maceration | O | Hematuria U06(3)  Bleeding A10(2)  Menstruation absent X05(4) | A,B,C,G,H,I,J,L,N |
| Xiuxianjv | *Spiraea salicifolia* L. | Rosaceae/20230601273/BY/W/shrub | Root/stem | Blend | T | Eye pain F01(3)  Headache N01(2)  Teeth ache DI9(4)  Cough R05(5) | A,B,C,D |
| Kuanruidijv | *Sanguisorba applanata* Yu et Li | Rosaceae/20230601008/BY/W/herb | Root | Burning/Decocting | O/T | Intoxication A86(3)  Acne S96(2) | A,B,C |
| Maomei | *Rubus parvifolius* L*.* | Rosaceae/20230511166/BY/W/shrub | Leaf | Decocting/alcohol maceration | O/T | Hemorrhoids K96 (3)  Dysentery NOS D73(2)  Vomiting blood D14 (9) | A,B,D,M,N |
| Niudiedu | *Rubus crataegifolius* Bge*.* | Rosaceae/20230608178/BY/W/shrub | Root/stem | Decocting | O | Impotence Y07(2)  Urination problems U05(2)  Viral hepatitis D72(3) | A,B,C,D |
| Yuli | *Cerasus japonica* (Thunb.) Lois. | Rosaceae/20230511302/BY/W/shrub | Fruit/seed | Decocting/blend | O | Swelling NOS A08(2)  Constipation D12(13)  Urination problems U05(2) | A,C,D,I,J,L |
| Shanlihong | *Crataegus pinnatifida* var. major N. E. Brown | Rosaceae/20230511302/BY/W/tree | Fruit | Decocting/blend | O/T | Dyspepsia D07 (18)  Diarrhea D11(6)  Abdominal pain D02 (5)  Vertiginous syndrome  H82 (4)  Hypertension K86(5)  Blurred vision F05(3)  Loss of appetite T03(15) | A,B,F,I,J,N |
| Longyacao | *Agrimonia pilosa* Ldb. | Rosaceae/20230526143/BY/W/herb | Whole plant | Decocting/blend/curshing | O/T | Gastroenteritis D73(5)  Vomiting blood D14(3)  Hemoptysis R24 (3)  Menstruation excessive X06 (2)  Diarrhea D11 (1) | A,B,C,D,H,I,N |
| Shanzha | *Crataegus pinnatifida* Bunge | Rosaceae/20240421213/BY/W/tree | Fruit/seed | Boiling | O | Hypertension K86(5)  Dyspepsia D07(13)  Diarrhea D11(7)  Abdominal pain D02 (4)  Loss of appetite T03(15) | A,B,C,D,I,J,K,N |
| Shuiyuhuajiu | *Sorbus alnifolia* (Sieb. et Zucc.) K. Koch | Rosaceae/20240421216/BY/W/tree | Fruit/bark | Decocting | O | Cough R05 (5)  Asthma R96(2)  Dyspepsia D07(4)  Nausea and vomiting D10(3)  Urinary frequency U02(3) | A,C,D |
| Weilingcao | *Potentilla chinensis* Ser*.* | Rosaceae/20230511250/BY/W/herb | Whole plant | curshing | O/ T | Gastroenteritis D73(3) | A,C,D,H,I,J,K,L,N |
| Yeqiangwei | *Rosa multiflora* Thunb. | Rosaceae/20230608287/BY/W/shrub | Flower | Decocting/  boiling | O | Menstruation absent X05(2)  Gastroenteritis D73(3)  Swelling NOS A08 (5) | A,B,C,D,N |
| Diyu | *Sanguisorba officinalis* L. | Rosaceae/20230601053/BY/W/herb | Root | Boiling/curshing/Vinegar scald | O | Burn/scald S14(1)  Hematuria U06(4)  Acne S96 (8) | A,B,C,D,H,J,K,L,M,N |
| Shanjingzi | *Malus baccata* (L.) Borkh. | Rosaceae/20230526173/BY/W/tree | Bark | Decocting/curshing | O | Diarrhea D11(4)  Nausea and vomiting D10(2)  Gastroenteritis D73(3) | A,B,C,D,N |
| Changyediyu | *Sanguisorba officinalis* var.  longifolia | Rosaceae/20230601008/BY/W/herb | Root | Boiling/curshing/Vinegar scald | O | Burn/scald S14 (2)  Hematuria U06(6)  Acne S96(1) | A,B,D,N |
| Ouli | *Prunus humilis* (Bge.) Sok. | Rosaceae/20230601016/BY/W/shrub | Fruit/seed | Decocting/curshing | O | Constipation D12 (9) | A,C,D,H,I,L,N |
| Duli | *Pyrus betulifolia* Bunge | Rosaceae/20230601314/BY/W/tree | fruit | Decocting | O | Constipation D12(2)  Swelling NOS A08(2)  Cough R05(7)  Gastroenteritis D73(3) | A,D,N |
| Shantao | *Amygdalus davidiana* (Carr.) C. de Vos | Rosaceae/20230601208/BY/W/tree | Fruit/seed | Decocting/curshing | O | Bruise/contusion S16 (8)  Constipation D12 (2)  Cough R05 (6) | A,D,I,N |
| Shemei | *Duchesnea indica* (Andr.) Focke | Rosaceae/20230601217/BY/W/herb | Whole plant | Decocting/curshing | O/ T | Gastroenteritis D73 (3)  Burn/scald S14 (4)  Sore throat R21 (3) | A,B,C,D,M,N |
| Mantuoluo | *Datura stramonium* Linn. | Solanaceae/20230825889/BY/W/herb | Leaf/Flower/seed | Decocting | O | Rheumatism L18 (3)  Cough R05 (6) | A,B,C |
| Yangjinhua | *Datura metel* L*.* | Solanaceae/20230825876/BY/W/herb | Leaf/flower/seed | Decocting | O | Abdominal pain D02 (3)  Burn/scald S14 (5)  Worms/other parasites D96 (4)  Pruritus S02 (2)  Swelling NOS A08(5)  Cough R05 (9) | A,B,C,J,M |
| Baiying | *Solanum lyratum* Thunb. | Solanaceae/20230608010/BY/W/vines | Whole plant | Crushing | T | Burn/scald S14 (3)  Jaundice D13 (4)  Rheumatism L18 (4)  Eczema S87 (5) | A,B,C,K,N |
| Longkui | *Solanum nigrum* L. | Solanaceae/20230608012/BY/W/herb | Whole plant | Decocting | O | Sore throat R21 (5)  Chronic bronchitis R79(7)  Bruise/contusion S16 (4) | A,B,C,G,K,M,N |
| Baoduocao | *Disporum sessile* D. Don | Colchicaceae/20230824361/BY/W/herb | Rhizome | Decocting | O/ T | Cough R05 (4) | A,B,C |
| Wanshouzhu | *Disporum cantoniense* (Lour.)  Merr. | Colchicaceae/20230824243/BY/W/herb | Root/stem | Decocting/  crushing | O/ T | Chronic bronchitis R79(4)  Sore throat R21 (5)  Cough R05 (3)  Dyspepsia D07(2) | A,B,C |
| Baijiang | *Patrinia scabiosaefolia* Link | Caprifoliaceae/20230825012/BY/W/herb | Whole plant | Decocting | O | Burn/scald S14(3)  Swelling NOS A08 (5)  Diarrhea D11 (3)  Appendicitis D88(2) | A,N |
| Rendong | *Lonicera japonica* Thunb. | Caprifoliaceae/20230601195/BY/W/shrub | Flower | Decocting/  crushing | O | Sore throat R21 (15)  Fever A03 (5)  Rheumatism L18(3) | A,B,C,F,H,I,J,L,M |
| Jindaihua | *Weigela florida* (Bunge) A. DC. | Caprifoliaceae/20230601196/BY/W/shrub | Flower | Decocting | O | Burn/scald S14(4)  Bruise/contusion S16(4) | A,C |
| Jinyinrendong | *Lonicera maackii* (Rupr.)  Maxim. | Caprifoliaceae/20230601286/BY/W/shrub | Flower | Decocting/raw | O | Sore throat R21 (8)  Fever A03 (4)  Rheumatism L18 (7) | A,B,C,N |
| Xiecao | *Valeriana officinalis* L. | Caprifoliaceae/20230601197/BY/W/herb | Root/stem | Decocting | O | Sleep disturbance P06 (8)  Menstruation absent X05(4)  Gastroenteritis D73 (4)  Bruise/contusion S16 (6) | A,B,C,K |
| Shechuang | *Cnidium monnieri* (L.) Cuss. | Apiaceae/20230608216/BY/W/herb | Fruit | Raw | O | Pruritus S02 (2)  Worms/other parasites D96 (7)  Eczema S87(5)  Impotence NOS Y07(6) | A,B,C,G,H,J,N |
| Gelouzi | *Carum carvi* L. | Apiaceae/20230608219/BY/W/herb | Root/stem | Decocting/  crushing | O | Dyspepsia D07(5)  Menstrual pain X02(5)  Flatulence D08(6) | A,B |
| Shuiqin | *Oenanthe javanica* (Bl.) DC. | Apiaceae/20230519351/BY/W/herb | Whole plant | Decocting/  juicing/crushing | O/ T | Fever A03 (4)  Sore throat R21(4)  Hematuria U06 (2)  Rheumatism L18 (3)  Urination problems U05(6) | A,B,C,N |
| Qieyi | *Torilis scabra* (Thunb.) DC. | Apiaceae/20230519371/BY/W/herb | Whole plant/fruit | Decocting | O/ T | Pruritus S02(3)  Worms/other parasites D96 (6)  Swelling NOS A08(2) | A,B,C |
| Guaiqin | *Angelica polymorpha* Maxim. | Apiaceae/20230519252/BY/W/herb | Root | Decocting/  crushing | O | Abdominal pain D02(2)  Rheumatism L18(3) | A,B,C,K |
| Sang | *Morus alba* L. | Moraceae/20230601200/BY/W/tree | Leaf/cortex | Decocting | O | Red eye F02(3)  Cough R05 (9)  Swelling NOS A08 (8)  Constipation D12(5)  Rheumatism L18(9) | A,B,C,F,G,H,I,J,M,N |
| Mensang | *Morus mongolica* (Bur.)  Schneid. | Moraceae/20230601230/BY/W/tree | Fruit/seed | Decocting/  juicing | O/T | Cough R05 (9)  Swelling NOS A08 (7)  Constipation D12(5)  Rheumatism L18(9) | A,B,C |
| Bianganbiaocao | *Scirpus planiculmis* Fr. Schmidt | Cyperaceae/20230601376/BY/W/herb | Tuber | Decocting | O | Menstruation absent X05 (5)  Dyspepsia D07(6) | A,B,G |
| Yabingtaicao | *Carex lanceolata* Boott var.  subpediformis Kukenth. | Cyperaceae/20230511280/BY/W/herb | Whole plant | Decocting | O | Fever A03 (4) | A,B,C |
| Yiguotaicao | *Carex neurocarpa* Maxim. | Cyperaceae/20230526293/BY/W/herb | Whole plant | Decocting | O | Fever A03 (3)  Urination problems U05(1) | A,B,C,M |
| Yixingsuocao | *Cyperus difformis* L. | Cyperaceae/20230526367/BY/W/herb | Whole plant | Decocting/  crushing | O | FlatulenceD08(4)  Menstruation absent X05(6) Urination problems U05(4) | A,B,C |
| Suocao | *Cyperus rotundus* L. | Cyperaceae/20230608202/BY/W/herb | Whole plant | Decocting/  crushing | O/ T | FlatulenceD08(2)  Rheumatism L18(3) | H,M |
| Baitan | *Symplocos paniculata* (Thunb.) Miq. | Symplocaceae/20230608137/BY/W/shrub | Root/stem | Raw | O | Intoxication A86 (4)  Measles A71 (6)  FlatulenceD08 (3) | A,C |
| Shanfan | *Symplocos sumuntia* Buch. Ham. ex D. Don | Symplocaceae/20230608128/BY/W/tree | Root/stem/flower/leaf | Juicing | O | Intoxication A86(5)  Eczema S87(6) | A,B |
| Chuixushanglu | *Phytolacca americana* L. | Symplocaceae/20230519034/BY/W/herb | Root | Decocting/  crushing | O/ T | Urination problemsU05(4)  Intoxication A86(2)  Constipation D12(11) | A,B, ,F,H,I,J,K,L,N |
| Duxingcai | *Lepidium apetalum* Willdenow | Brassicaceae/20230519057/BY/W/herb | Whole plant | Raw/Decocting | O | Asthma R96(6)  Cough R05(8) | A,B,C,F,G,I,K |
| Beimeiduxingcai | *Lepidium virginicum* Linnaeus | Brassicaceae/20230825331/BY/W/herb | Whole plant | Decocting | O | Cough R05(7) | A,B,C,N |
| Fenghuacai | *Rorippa globosa* (Turcz.) Hayek. | Brassicaceae/20230608069/BY/W/herb | Whole plant | Decocting/  crushing | O/ T | Sore throat R21(6) | A,B,C,N |
| Ji | *Capsella bursa-pastoris* (L.)  Medic. | Brassicaceae/20230421186/BY/W/herb | Whole plant | Decocting | O | Menstruation excessive X06 (6)  Bleeding A10(3) | A,C,G,M |
| Suimiqi | *Cardamine hirsuta* L. | Brassicaceae/20240421235/BY/W/herb | Whole plant | Decocting/  crushing | O/ T | Eczema S87(4)  Bleeding A10(5) | A,C |
| Bonianhao | *Descurainia sophia* (L.) Webb  ex Prantl | Brassicaceae/20240421022/BY/W/herb | Fruit/seed | Decocting | O | Urination problemsU05(4)  Cough R05(8) | A,B,C,F,G,H,I,L,M,N |
| Shi | *Diospyros kaki* Thunb. | Ebenaceae/20230825225/BY/W/tree | Persistent calyx | Decocting | O | Cough R05 (3)  Nausea and vomiting D10(5) | A,B,C,I,J |
| Shanjiu | *Allium senescens* L. | Amaryllidaceae/20230825888/BY/W/herb | Whole plant | Decocting/  crushing | O/T | Dyspepsia D07(2) | A,B,C,N |
| Xiebai | *Allium macrostemon* Bunge | Amaryllidaceae/20230526270/BY/W/herb | Root/stem |  | O/T | FlatulenceD08(5) | A,B,C,I,J,N |
| Haiershen | *Pseudostellaria heterophylla*  (Miq.)Pax | Caryophyllaceae/20230601346/BY/W/herb | Tuber | Decocting | O | Dyspepsia D07(6) | A,B,C |
| Changruishitouhua | *Gypsophila oldhamiana* Miq. | Caryophyllaceae/20230601315/BY/W/herb | Root/stem | Decocting | O | Abdominal pain epigastric D02(5)  Bleeding A10(7) | A,B,C,K,M,N |
| Echangcai | *Myosoton aquaticum* (L.)  Moench | Caryophyllaceae/20240421063/BY/W/herb | Whole plant | Decocting/  crushing | O/T | Rheumatism L18(3)  Scabies S72(2) | A,B,M,N |
| Shizhu | *Dianthus chinensis* L. | Caryophyllaceae/20230519224/BY/W/herb | Whole plant | Decocting | O | Urination problems U05(5)  Menstruation absent X05(3) | A,B,C,I,K,L,N |
| Mailancai | *Vaccaria hispanica* (Mill.)  Rauschert | Caryophyllaceae/20230519314/BY/W/herb | Seed | Decocting | O | Menstrual pain X02 (5)  Menstruation absent X05 (12)  Lactation symptom W19 (13) | A,B |
| Jianyingnvloucai | *Silene firma* Sieb. et Zucc. | Caryophyllaceae/20230519340/BY/W/herb | Whole plant | Decocting/  crushing | O/T | Intoxication A86(5)  Rheumatism L18(6) | A,B,C |
| Nvloucai | *Silene aprica* Turcz. ex Fisch.  et Mey. | Caryophyllaceae/20230526181/BY/W/herb | Whole plant | Decocting/  crushing | O/T | Menstruation absent X05(6)  Intoxication A86(6) | A,B,K |
| Qumai | *Dianthus superbus* L. | Caryophyllaceae/20230601502/BY/W/herb | Above ground | Decocting/  crushing | O/T | Menstruation absent X05 (6) Urination problems U05(7) | A,B,C,J,K,N |
| Junqianzi | *Diospyros lotus* L. var. lotus | Ebenaceae/20230601435/BY/W/tree | Fruit | Decocting | O | Impotence NOS Y07 (6)  Constipation D12 (3)  Phlegm R25(3)  Cough R05 (3) | A,B,C,N |
| Suanzao | *Ziziphus jujuba* Mill. var.  spinosa (Bunge) Hu ex H. F.  Chow | Rhamnaceae/20230601234/BY/W/shrub | Fruit/seed | Decocting/  crushing/raw | O/T | Heartburn D03(6)  Sleep disturbance P06  (6) | A,B,F,H,J,L,M |
| Zao | *Ziziphus jujuba* Mill. | Rhamnaceae/20230601372/BY/W/tree | Fruit | Decocting | O | Heartburn D03(4)  Gastroenteritis D73(1)  Flatulence D08 (5) | A,C,H,J |
| Yuanyeshuli | *Rhamnus globosa* Bunge | Rhamnaceae/20230511305/BY/W/shrub | Bark/stem/Leaf | Decocting | O | Scabies S72(2) | A,B,C |
| Shuli | *Rhamnus davurica* Pall | Rhamnaceae/20230511353/BY/W/shrub | Fruit | Decocting/  crushing | O/T | Hemorrhoids K96 (1) Eczema S87 (4)  Constipation D12 (3) | A,B,C |
| Chaoxianshuli | *Rhamnus koraiensis* Schneid. | Rhamnaceae/20230511605/BY/W/shrub | Fruit | Decocting/  crushing | O/T | Eczema S87(4)  Constipation D12(3) | A |
| Shuyu | *Dioscorea oppositifolia* L. | Dioscoreaceae/20230801234/BY/W/vines | Tuber | Decocting | O | Musculoskeletal diseases L99(6) | A,C,I,K,L,N |
| Chuanlongshuyu | *Dioscorea nipponica* Makino | Dioscoreaceae/20230801260/BY/W/vines | Root/stem | Decocting/  crushing | O/T | Menstruation absent X05(5)  Cough R05(4)  Gastroenteritis D73(3)  Flatulence D08(3)  Headache N01(1) | A,B,C, ,L,N |
| Heisong | *Pinus thunbergii* Parlatore | Pinaceae/20230601081/BY/W/tree | Flower | Decocting | O | Hemoptysis R24(2)  Scabies S72(2)  Bleeding A10(3) | A,C |
| Maweisong | *Pinus massoniana* Lamb. | Pinaceae/20240421159/BY/W/tree | Flower | Decocting/  crushing | O | Cough R05(3)  Constipation D12(2)  Eczema S87(3)  Bleeding A10(3)  Scabies S72(2) | A,B,C |
| Luoyesong | *Larix gmelinii* (Ruprecht)  Kuzeneva | Pinaceae/20240421126/BY/W/tree | Flower | Decocting/  crushing | O/T | Bleeding A10(1)  Eczema S87(2)  Scabies S72(4)  Skin injury S19(1) | A,C |
| Chisong | *Pinus densiflora* Sieb. et Zucc. | Pinaceae/20230526030/BY/W/tree | Flower | Decocting/  crushing | O/T | Measles A71(2)  Intoxication A86(2)  Constipation D12(2)  Cough R05(1) | A,B,C,J,N |
| Luyao | *Smilacina japonica* A. Gray | Asparagaceae/20230526034/BY/W/herb | Root/stem | Decocting/  crushing | O/T | Kidney symptom U14(4)  Menstruation absent X05 (5)  Rheumatism L18(7) | A |
| Maidong | *Ophiopogon japonicus* (Linn. f.) Ker-Gawl. | Asparagaceae/20230601160/BY/W/herb | Tuber | Decocting/  crushing | O/T | Fever A03(8)  Cough R05(4)  Constipation D12 (6)  Sleep disturbance P06(8) | A,B,C,I,J |
| Yuzhu | *Polygonatum odoratum* (Mill.)  Druce | Asparagaceae/20240421503/BY/W/herb | Rhizome | Decocting/  crushing | O/T | Cough R05 (9)  Phlegm R25(10)  Loss of appetite T03(8)  Sore throat R21(4) | A,B,C,J,K,L,N |
| Mianzaoer | *Scilla scilloides* (Lindl.) Druce | Asparagaceae/20240421603/BY/W/herb | Bulb/whole plant | Decocting/  crushing | O/T | Bruise/contusion S16(4)  Heart disease K84 (3)  Swelling NOS A08(5) | A,K,N |
| Linglan | *Convallaria majalis* Linn. | Asparagaceae/20240421506/BY/W/herb | Whole plant | Decocting/  crushing | O/T | Heart disease K84 (3)  Rheumatism L18 (3)  Swelling NOS A08(5) | A,B,C |
| Huangjing | *Polygonatum sibiricum* | Asparagaceae/20240421203/BY/W/herb | Rhizome | Decocting | O | Cough R05(9)  Loss of appetite T03(4)  Hemoptysis R24(5) | A,B,C,J,K,L,N |
| Tianmendong | *Asparagus cochinchinensis*  (Lour.) Merr | Asparagaceae/20230519173/BY/W/herb | Tuber | Decocting | O | Sore throat R21 (5)  Constipation D12(6)  Cough R05 (6)  Phlegm R25(8) | A,B,C |
| Nanyudai | *Asparagus oligoclonos* Maxim. | Asparagaceae/20230519176/BY/W/herb | Root | Decocting | O | Fever A03(7)  Chronic bronchitis R79(9)  Asthma R96(5)  Sore throat R21(6) | A,B,C,N |
| Shanmaidong | *Liriope spicata* (Thunb.) Lour. | Asparagaceae/20230519132/BY/W/herb | Tuber | Decocting | O | Cough R05(5)  Sore throat R21(9)  Sleep disturbance P06(9)  Constipation D12(6) | A,B,C,I,J,K |
| Dongbeinanxing | *Arisaema amurense* Maxim*.* | Araceae/20230519309/BY/W/herb | Root/stem | Crushing | T | Cough R05(11)  Phlegm R25(10)  insect bite S12(5) | A,C, |
| Tougucao | *Phryma leptostachya s*ubsp.  asiatica (Hara) Kitamura | Phrymaceae/20230519328/BY/W/herb | Whole plant | Decocting/  crushing | O/T | Rheumatism L18(11) Bruise/contusionS16 (5)  Menstruation absent X05(6) | A,B,K,N |
| Baidu | *Euonymus maackii* Rupr. | Celastraceae/20230519337/BY/W/tree | Root/bark | Decocting | O | Rheumatism L18(7)  Kidney symptom U14(3)  Menstruation absent X05 (8) | A,B,K,N |
| Nansheteng | *Celastrus orbiculatus* Thunb. | Celastraceae/20230526175/BY/W/vines | Root | Decocting/  crushing | O/T | Rheumatism L18(2)  Toothache D19(4)  Headache N01(2)  Menstrual pain X02(3)  Menstruation absent X05(5) | A,B,C,K,N |
| Weimao | *Euonymus alatus* (Thunb.) Sieb | Celastraceae/20240421245/BY/W/shrub | Root | Decocting/  crushing | O/T | Bruise/contusion S16(3)  Menstruation absent X05(2) | A,B,C,N |
| Fufangteng | *Euonymus fortunei* (Turcz.)  Hand.-Mazz. | Celastraceae/20240421071/BY/W/shrub | Leaf | Decocting/  crushing | O/T | Hemoptysis R24(2)  Menstruation absent X05(4)  Bruise/contusion S16(2) | A,B,C,M |
| Chuisiweimao | *Euonymus oxyphyllus* Miq. | Celastraceae/20240421061/BY/W/shrub | Root/stem/cortex | Decocting/  crushing | O/T | Rubella A74(3)  Eczema S87(6)  Intoxication A86(2)  Rheumatism L18(6)  Menstruation absent X05 (3) | A,B,C |
| Yuanbaoqi | *Acer truncatum* Bunge | Sapindaceae/20230421091/BY/W/tree | Bark | Decocting | O | Rheumatism L18(2)  Musculoskeletal disease L99(2) | A,B,C,KN |
| Jiami | *Viburnum dilatatum* Thunb. | Adoxaceae/20240421161/BY/W/shrub | root/stem | Decocting/  crushing | O/T | Fever A03 (3)  Bruise/contusion S16 (2) | A,B,C |
| Yichangjiami | *Viburnum erosum* Thunb. | Adoxaceae/20230421181/BY/W/shrub | Root/stem | Decocting | O | Rheumatism L18(1)  Mouth symptom D20(4) | A,B |
| Jishutiao | *Viburnum opulus* subsp.  calvescens (Rehder) Sugimoto | Adoxaceae/20240421068/BY/W/shrub | Fruit/seed | Decocting/  crushing | O/T | Rheumatism L18 (4)  Swelling NOS A08(2)  Rubella A74(2) | A,N |
| Ciqiu | *Kalopanax septemlobus* (Thunb.) Koidz. var. septemlobus | Araliaceae/20240421146/BY/W/tree | Bark | Decocting | O/T | Rheumatism L18(2)  Bruise/contusion S16 (5) | A,B,C |
| Li | *Chenopodium album* L. | Amaranthaceae/20240421130/BY/W/herb | Whole plant | Decocting | O/T | Diarrhea D11(2)  Abdominal pain D02 (3)  Pruritus S02(3) | A,B,C,G |
| Niuxi | *Achyranthes bidentata* Blume  var. bidentata | Amaranthaceae/20230601180/BY/W/herb | Root/stem | Decocting/  crushing | O/T | Kidney symptom U14(6)  Menstruation absent X05(9)  Urination problems U05(3) | A,B,C,H,I,J,L,N |
| Lianzicao | *Alternanthera sessilis* (L.) DC. | Amaranthaceae/20230601231/BY/W/herb | Whole plant | Decocting/  juicing | O/T | Dysentery NOS D73(4)  Vomiting blood D14 (3)  Sore throat R21(3)  Urination problems U05(5) | A,B,C |
| Xiangpu | *Typha orientalis* Presl | Typhaceae/20230526255/BY/W/herb | Pollen | Decocting/  crushing | O/T | Bleeding A10(4)  Gonorrhea female X71 (4)  Gonorrhea male Y71(3) | A,C,K,N |
| Soushu | *Deutzia scabra* Thunb | Hydrangeaceae/20230526263/BY/W/shrub | Root/leaf/fruit | Decocting/  juicing | O/T | Urination problems U05(8)  Malaria A73(2)  Intoxication A86(5)  Musculoskeletal disease L99(4) | A |
| Qianniu | *Ipomoea nil* (Linnaeus) Roth | Convolvulaceae/20230608189/BY/W/herb | Seed | Decocting | O | Constipation D12(9)  Phlegm R25(3)  Worms/other parasites D96(2) | A,B,G,H,I,J,N |
| Yuanyeqianniu | *Ipomoea purpurea* Lam*.* | Convolvulaceae/20230519330/BY/W/herb | Seed | Decocting/  crushing | O/T | Constipation D12(9)  Flatulence D08(4)  Swelling NOS A08(7)  Worms/other parasites D96(4) | A,C,G,H,I,J,L,N |
| Dayezhuma | *Boehmeria longispica* Steud. | Urticaceae/20230519439/BY/W/shrub | Leaf | Decocting/  crushing | O/T | Fever A03(3)  Swelling NOS A08(2) | A |
| Xiaochima | *Boehmeria spicata* (Thunb.)  Thunb. | Urticaceae/20230519843/BY/W/herb | Root/stem | Decocting/  crushing | O/T | Bruise/contusion S16 (3)  Swelling NOS A08(2)  Measles A71(1)  Menstruation absent X05(2) | A,B,C |
| Yatuocao | *Commelina communis* L. | Commelinaceae/20230601279/BY/W/herb | Stem leaf | Decocting/  crushing | O/T | Fever A03 (3)  Sore throat R21(4) | A,B,C,H,I,J,L,N |
| Hanliu | Salix matsudana Koidz | Salicaceae/20230421078/BY/W/tree | Bark | Decocting | O | Fever A03 (2)  Injuries A80(3)  Toothache D19(5)  Urination problems U05(2) | A,B,C,G,N |
| Yexiazhu | *Phyllanthus urinaria* L. | Phyllanthaceae/20230601243/BY/W/herb | Whole plant | Decocting/  crushing | O/T | Swelling NOS A08(3)  Fever A03(2)  Gastroenteritis D73(5) | A,B,C |
| Yinxing | *Ginkgo biloba* L. | Ginkgoaceae/20230601295/BY/W/tree | Fruit/seed/leaf | Decocting | O | Urination problems U05(2)  Asthma R96(4)  Urinary frequency U02 (5)  Menstruation absent X05 (2)  Cough R05(9)  Bruise/contusion S16(2) | A,B,C,H,I,J,M,N |
| Zijin | *Corydalis edulis* Maxim. | Papaveraceae/20230601273/BY/W/herb | Whole plant | Decocting/  crushing | O/T | Fever A03(3)  Intoxication A86(3)  Eczema S87(2)  Cough R05(5)  Impotence Y07(1) | A,B,C,I |
| Xiaohuangzijin | *Corydalis raddeana* Regel | Papaveraceae/20230601126/BY/W/herb | Whole plant | Decocting/  crushing | O/T | Fever A03(3)  Intoxication A86(1)  Worms/otherparasitesD96(3) Pruritus S02(2) | A,B,C |
| Langyu | *Ulmus parvifolia* Jacq | Ulmaceae/20230511127/BY/W/tree | Root/bark | Decocting/  crushing | O/T | Toothache D19(3)  Urination problems U05(2) Hematuria U06(3)  Dysentery NOS D73(4)  Burn/Scald S14(2)  Haemorrhoids K96(1) | A,B,C,N |
| Yushu | *Ulmus chenmoui* Cheng | Ulmaceae/20240421299/BY/W/tree | Fruit/seed, cortex/leaf | Decocting/  crushing | O/T | Swelling NOS A08(4)  Urination problems U05(6)  Sleep disturbance P06(3)  Heat exhaustion A88(6)  Cough R05(1)  Phlegm R25(4) | A,B,C,G,N |
| Daguoyu | *Ulmus macrocarpa* Hance | Ulmaceae/20240411039/BY/W/tree | Fruit/seed | Decocting/  crushing | O/T | Phlegm R25(2)  Cough R05 (5)  Worms/other parasites D96(2) | A,B,C,N |
| Shegan | *Belamcanda chinensis* (L.)  Redouté. | Iridaceae/20230601219/BY/W/herb | Rhizome | Decocting | O | Upper respiratory infectionI acute R74(6)  Phlegm R25(2)  Sore throat R21(4)  Constipation D12(1)  Urination problems U05(4) | A,B,C,J,N |
| Qinghuajiao | *Zanthoxylum schinifolium* Sieb.  et Zucc. | Rutaceae/20230601139/BY/W/shrub | Root/leaf/fruit | Decocting | O/T | Worms/other parasites D96(2)  Hypertension K86(3)  Loss of appetite T03(2) | A,B,C,N |
| Huajiao | *Zanthoxylum bungeanum*  Maxim. | Rutaceae/20230601085/BY/W/tree | Fruit/seed | Decocting | O/T | Cough R05(1)  Abdominal pain D02(5)  Diarrhea D11(2)  Nausea and vomiting D10(5)  Worms/other parasites D96(5)  Pruritus S02(2)  Toothache D19(6)  Dysentery NOS D73(7)  Hernia D91 (5) | A,B,C,H,J,M,N |
| Sanyawuyao | *Lindera obtusiloba* Bl. Mus.  Bot. var. obtusiloba | Lauraceae/20230601076/BY/W/tree | Bark | Decocting/  crushing | O/T | Contusion S16(2)  Injuries A80(4) | A,B,C |
| Shanhujiao | *Lindera glauca* (Sieb. et Zucc.) Bl | Lauraceae/20230601325/BY/W/tree | Root/fruit | Decocting | O | Abdominal pain D02(2)  Stomach function disorder D87(3)  Rheumatism L18(2)  Swelling NOS A08(3)  Contusion S16(4)  Injuries A80 (3)  Burn/Scald S14 (2)  Chronic bronchitis R79(2) | A,B,C |
| Banzhongcao | *Bothriospermum chinense* Bge. | Boraginaceae/20230621061/BY/W/herb | Whole plant | Decocting/  crushing | O/T | Dermatitis S86(2)  Pruritus S02(3)  Eczema S87(4)  Impotence NOS Y07 (2) | A,B,C |
| Duobaobanzhongcao | *Bothriospermum secundum*  Maxim. | Boraginaceae/20230526060/BY/W/herb | Whole plant | Decocting | O | Fever A03 (2)  Swelling NOS A08(2)  Eczema S87(2) | A,B |
| Heshi | *Lappula myosotis* Moench | Boraginaceae/20230526070/BY/W/herb | Fruit | Decocting | O | Worms/other parasites D96(2) | A,B,G,H |
| Fudicai | *Trigonotis peduncularis* (Trev.) Benth. ex Baker et Moore | Boraginaceae/20230421072/BY/W/herb | Whole plant | Decocting/  crushing | O/T | Abdominal pain D02(3)  Swelling NOS A08(2) | A,B,C,G,M,N |
| Zicai | *Lithospermum erythrorhizon*  Sieb. et Zucc. | Boraginaceae/20230519326/BY/W/herb | Root/stem | Decocting/  juicing | O/T | Jaundice D13(3)  Eczema S87(2)  Constipation D12(4)  Hematuria U06(2)  Burn/Scald S14 (3)  Erysipelas S76(4) | A,B,C,J |
| Ziqi | *Osmunda japonica* Thunb. | Osmundaceae/20230519436/BY/W/herb | Stem leaf | Decocting/  crushing | O/T | Fever A03 (5)  Bleeding A10(2)  Worms/other parasites D96(2) | A,B,C |
| Zishu | *CatalpaovataG.* Don. | Bignoniaceae/20230519264/BY/W/tree | Seed/bark | Decocting/crushing/juicing | O/T | Jaundice D13(2)  Eczema S87(2)  Nausea and vomiting D10(3)  Worms/other parasites D96(1)  Pruritus S02(1)  Scabies S72(1) | A,C |

^a^ The voucher specimens are presented in the herbarium of the School of Pharmacy, Binzhou Medical University.

^b^ Habitat: w, wild.

^c^ R, route of administration: O, oral; T, topical.

^d^ Materia medica records: A: Editorial Committee of Flora of China (1989-2013); B: Fu, 2012; C: Institute of Botany, Chinese Academy of Sciences (1982); D: Li et al., 2023; E: Guan et al., 2016; F: Yang et al., 2020; G: Gao et al., 2017; H: Zhang et al., 2020; I: Gao et al., 2019; J: Chinese Pharmacopoeia Commission (2015); K: Yang, 2024;L: Yang et al., 2023; M: Lin et al., 2021; N: Yan, et al., 2023.

**Including the reference list at the**

Chinese Pharmacopoeia Commission, (2015). Pharmacopoeia of the People’s Republic of China. China Medicinal Science Press, Beijing, China.

Editorial Committee of Flora of China, (1989-2013). Flora of China. Science Press, Beijing, China.

Fu, L.G., Chinese Higher Plants. (2012). Qingdao Publishing House. Qingdao, China.

Gao, F., Zhang, T.T., Yue, G.Y., Sun, Z.Y., (2019). Investigation on Medicinal Plant Resources in Tengzhou City, Shandong Province. Journal of Shandong University of Traditional Chinese Medicine 43(06), 603-610.

Gao,Y.J., Zhou, F.Q., Han, L.N., Zhang, X.Y., Shi, G.Y., (2017). Investigation on the Source of Medical Plants in Kenli County ShandongProvince. Research and Practice of Modern Chinese Medicine 31(02), 17-21.

Guan, R.W., Lu, J.X., Wang, M., Fu, Q.Z., Guo, R.Q., Hu, C.M., et al. (2016). Investigation on Resources Diversity of Legumes Medical Plants in Linquof Shandong Province. Wild Plant Resources in China 35(05), 70-73.

Institute of Botany, Chinese Academy of Sciences. (1982). Illustration of Higher Plants in China - Volume I. Science Press, Beijing, China.

Li, B., Chen, G.Y., Wei, G.D., Liu, H., (2023). Study and Investigation on Wild Plant Resource of Rosaceae in Yantai CityShandong Province. Wild Plant Resources in China 42(02), 103-109.

Lin, Y., Wang, S.P., Zhang, J.Y., Zhuo, Z.Y., Li, X.R., Zhai, C.J., et al. (2021). Ethnobotanical survey of medicinal plants in Gaomi, China. Journal of ethnopharmacology 265, 113228. <https://doi.org/10.1016/j.jep.2020.113228>

Yang, W.H., (2024). Survey and Protection of Medicinal Plant Resources in Boshan DistrictZibo City. Wild Plant Resources in China 43(02), 114-121+126.

Yang, X.T., Ran, Z.F., Zhang, Q.M., Xu, Z.X., Sun, H., Fu, B., et al. (2020). Survey and Analysis of Chinese Materia Medica Resources in ChangleCounty of Shandong Province. Journal of Shandong University of Traditional Chinese Medicine 44(04), 434-439.

Yang, Y.P., Wang, X.S., Wei, G.D., Liu, B., (2023). Study on Diversity of Medicinal Plant Resources in Qixia City,Shandong Province. Wild Plant Resources in China 42(01), 93-99+102.

Zhang, Y.F., Wen, X.S., Cheng, H.N., Zhao, Y., (2020). Study on the Diversity of Medicinal Plant Resources in Tai'erzhuangDistrict, Shandong Province. Traditional Chinese Medicinal Herbs 43(08), 1854-1859.

Yan, M., Li, Z.Q., Zhang, C.T., (2023). Investigation and Analysis of Medicinal Plant Resources in Changqing District of Jinan City, Shandong Province. Shandong University of Traditional Chinese Medicine.
